# Modified Activation Process for Supercapacitor Electrode Materials from African Maize Cob

**DOI:** 10.3390/ma13235412

**Published:** 2020-11-27

**Authors:** Moses Kigozi, Ravi Kali, Abdulhakeem Bello, Balaji Padya, Godwin Mong Kalu-Uka, John Wasswa, Pawan Kumar Jain, Peter Azikiwe Onwualu, Nelson Yaw Dzade

**Affiliations:** 1 Department of Materials Science and Engineering, African University of Science and Technology, Km 10 Airport Road, Galadimawa, P.O. Box 681 Garki Abuja, Nigeria; abello@aust.edu.ng (A.B.); gkaluuka@aust.edu.ng (G.M.K.-U.); aonwualu@aust.edu.ng (P.A.O.); 2 Department of Chemistry, Faculty of Science and Education, Busitema University, Tororo P.O. Box 236, Uganda; 3 Centre for Carbon Materials, International Advanced Research Centre for Powder Metallurgy & New Materials (ARCI), Balapur P.O. Hyderabad 500 005, India; nanoravi09@gmail.com (R.K.); balajipadya@gmail.com (B.P.); pkjain@arci.res.in (P.K.J.); 4 Department of Mechanical Engineering, Alex Ekwueme Federal University, Ndufu-Alike, Ebonyi State, P.O. Box 1010, Abakaliki, Nigeria; 5 Department of Chemistry, College of Natural Sciences, Makerere University, P.O. Box 7062 Kampala, Uganda; jnwasswa@chemistry.mak.ac.ug; 6 School of Chemistry, Cardiff University, Main Building, Park Place, Cardiff CF10 3AT, UK

**Keywords:** biomass, acid-activation, oxidation, specific capacitance, electrode material

## Abstract

In this work, African maize cobs (AMC) were used as a rich biomass precursor to synthesize carbon material through a chemical activation process for application in electrochemical energy storage devices. The carbonization and activation were carried out with concentrated Sulphuric acid at three different temperatures of 600, 700 and 800 °C, respectively. The activated carbon exhibited excellent microporous and mesoporous structure with a specific surface area that ranges between 30 and 254 m^2^·g^−1^ as measured by BET analysis. The morphology and structure of the produced materials are analyzed through Field Emission Scanning Electron Microscopy (FESEM), Fourier Transform Infrared Spectroscopy (FTIR), X-Ray Diffraction (XRD), Boehm titration, X-ray Photoelectron Spectroscopy (XPS) and Raman Spectroscopy. X-ray photoelectron spectroscopy indicates that a considerable amount of oxygen is present in the materials. The functional groups in the activated carbon enhanced the electrochemical performance and improved the material’s double-layer capacitance. The carbonized composite activated at 700 °C exhibited excellent capacitance of 456 F g^−1^ at a specific current of 0.25 A g^−1^ in 6 M KOH electrolyte and showed excellent stability after 10,000 cycles. Besides being a low cost, the produced materials offer good stability and electrochemical properties, making them suitable for supercapacitor applications.

## 1. Introduction

Electrochemical capacitors (supercapacitors) have recently received significant scientific and technological attention because of their exciting possibilities as energy storage devices. Supercapacitors, however, have low energy density (<10 Wh kg^−1^) compared to other storage devices such as batteries, limiting their widespread application in hybrid electric vehicles, backup power sources, renewable energy systems and industrial energy management [1]. Several research efforts have been made to improve the energy density of supercapacitors by developing nanostructured active electrode materials with suitable surface area, porosity and morphology to increase energy density without sacrificing their intrinsic high-power density and cycle life [2]. This can also be achieved by exploring electrolytes with large potential windows such as ionic or organic electrolytes. Depending on the charge storage mechanisms of the active materials used, the electrochemical systems (EC) can be generally classified into two types, namely electric double-layer capacitors based on ion adsorption (EDLCs mainly carbonaceous material with a high surface area) and pseudocapacitors (including redox material such as transition metal oxides and conducting polymers) [3]. Pseudocapacitors have been shown to exhibit much higher capacitance than the EDLCs and are either used to complement EDLCs to improve the power and energy densities [4].

An electrode is considered the backbone of an electrical double layer capacitor because supercapacitors’ overall performance and stability depend upon the materials used as electrodes. Many electrode materials are used in supercapacitors; each material demonstrating a unique capacitance behavior and performance. For electrode fabrication, materials that possess large specific surface areas and pore volume like mesoporous and microporous materials are attractive. Several research groups have attempted to fabricate electrodes from different carbon sources [5,6]. Despite all these efforts, activated carbon (AC) is still the choice material used in the industry due to its high specific surface area (SSA), low cost, good electrochemical performance and stability [7]. Different carbonaceous electrode materials are used in supercapacitors (SC) because of their high surface area, porosity and surface functional groups. For example, carbon composites [8], carbide-derived carbon [9], graphene and its derivatives [9], carbon aerogels [10,11] among others. AC derived materials are believed to have higher porosity, high surface area, high chemical and physical stability and high packing density [12,13]. Commercially available ACs are synthesized from different biomass due to their low cost of production and environmental friendliness. The porous nature of AC materials is advantageous in SC application, with the microporous and microporous being more influential for charge transfer processes. The high capacitance values are primarily due to the surface area of the material and electrolytes’ stability [14].

Biomass is a viable and sustainable source of materials and alternative to fossil fuel in energy applications and presently provide ~14% of the total energy consumed and ~35% for cooking and heating in third world countries, especially Africa [15]. Bio-inspired materials as electrodes for supercapacitor applications have become attractive because biomass resources are readily available, abundant, naturally renewable, eco-friendly [16,17]. Slow progress has been made so far towards developing AC as electrodes for supercapacitor from biomass materials. Biomass such as maize corncobs, rice husks, coconut shells and sugarcane bagasse, to mention but a few, have been explored to produce porous activated carbon for industrial applications [18,19,20,21,22,23,24,25,26,27]. Activated carbons with varying surface areas are have being used for industrial applications as energy storage materials, removal of toxic compounds, purification and separation in liquids and gases, catalysts or catalysts support [18,28,29,30], reduction in CO_2_ [31], removal of dyes and odor [30]. Other ecological biomass materials such as eggshell [32], wood sawdust [23], pistachio nutshells [33], cigarette filter [34], sunflower seed shell [35] and rice husk [36] have also been investigated as possible carbon sources for SC applications.

Zea Mays, commonly known as maize or corn, is the most widely cultivated cereal grain food worldwide. Maize cultivation is widespread, particularly in the Eastern, Western, Southern and central parties of Africa. This plays a significant role in most homes as support for food and income-earning for the rural economy. The production yield per hectare (YPH) (tons/hectare/year) was estimated at approximately 32 YPH by 2020, which is greater than the USA, China and Brazil combined at 30 tons/hectare/year [37]. Normally, the maize corn cobs are treated as agricultural crop residue/waste due to harvesting where only the grains are taken for further processing. The cobs are disposed-off in the farm, power plant sites and others are burnt to ashes, causing environmental pollution.

Many research efforts on biomass have been reported; for example, Ding et al. reported a sodium ion capacitor with the cathode and anode based on peanut shell nanosheets carbon with a specific surface area (SSA) of 2396 m^2^g^1^, yielding specific capacity of 161 mAhg^−1^ at 0.1 A g^−1^ and 73 mAh g^−1^ at 25.6 A g^−1^ [38]. The same research group reported hemp bast fibers, which exhibited SSA of 2287 m^2^ g^−1^ and a specific capacitance (CSP) of 106 Fg^−1^ at 10 Ag^−1^ in ionic liquids [39]. Seaweed was also pyrolyzed at different temperatures under a nitrogen atmosphere with SSA of 1300 m^2^ g^−1^, resulting in a high volumetric capacitance [40]. Dead leaves were pyrolyzed by Mandakini et al. [24] to produce carbon materials, exhibiting an SSA of 1230 m^2^ g^−1^ and a CSP of 400 F g^−1^ in 1 M H_2_SO_4_ electrolyte. Human hair was also explored as a source of carbon for SC and symmetric cell exhibited a CSP of 340 Fg^−1^ and 126 Fg^−1^ both in the alkaline electrolyte (6 M KOH) and organic electrolyte (1 M LiPF6 in EC/DEC) respectively, at 1 Ag^−1^ with excellent cycling ability [41]. Carbon synthesized from corncob has been used as an electrode in an electrochemical double-layer capacitor (EDLC) by Ghosh et al. [42]. The material produced was pre-treated with 5% ZnCl_2_ using Co as catalyst pyrolyzed at 700 °C and a fabricated device exhibited a specific capacitance of 270 F/g at a scan rate of 5 mV/s. Similarly, corncob-derived carbon with hierarchical porosity was reported [43]. The carbon produced displayed the special biogenetic textures of corncob benefiting from proper activation process; the carbon materials exhibited excellent electrical conductivity and high specific capacitance (293 Fg^−1^ at 1 Ag^−1^). Corncob lignin-based porous carbon modified reduced graphene oxide film was also reported for a flexible supercapacitor electrode [44]. The assembled supercapacitor had the advantages of flexible, lightweight, low price and environment friendly, which can achieve a high specific capacitance of 324.5 mF/cm^2^ at 0.2 mA/cm^2^ and 91.8% capacitance retention after 1000 charging/discharging cycles. Furthermore, a two-step method for preparing high-performance corncob-based activated carbons as supercapacitor electrodes was reported using ammonium chloride as a pore-forming additive followed by carbonization. The device fabricated had a good rate performance with a capacitance of 175 Fg^−1^ at 0.5 Ag^−1^ and good cycling stability after a 10,000 charge/discharge test [45].

However, there is limited report in the literature regarding the synthesis of activated carbon from maize cobs and its electrochemical performance as an electrode material has not been systematically. This present research uses the liquid-phase oxidation process and gas phase method to produce enhanced porous carbon from maize cobs at three different temperatures of activation after treatment with concentrated sulphuric acid (conc’n H_2_SO_4_). The use of sulphuric acid has been shown to effectively hydrolyze and oxidize materials introducing a sufficient number of functional groups on the surface of carbon, which include carboxyl, lactone, phenolic, sulphur groups, among others [31]. These functional groups are demonstrated to improve the efficiency of the non-faradaic and faradaic behavior in electron and charge transfer processes.

## 2. Experiments

### 2.1. Preparation of Activated Carbon

The activated carbons (ACs) were prepared from maize cobs (MC) as follows; the MC was obtained from farmlands within the African University of Science and Technology (AUST) Abuja, Nigeria. The MC was cleaned and sized (~3 cm), then dried in an oven at 110 °C for 48 h. The dried samples were grounded and sieved to fraction using 1.0 mm sieve. The MC powder was functionalized by chemical treatment using 14% *wt*/*v* conc’n H_2_SO_4_. The functionalized MC was left to stand for 48 h in a fume hood, then washed with DI water until a pH 6.5 was achieved. The sample was then dried in an electric oven at 120 °C for 18 h. The dried sample was divided into three portions. These portions were activated at three (3) different temperatures in a furnace under a nitrogen atmosphere with a 300 mL/min flow rate with a ramped temperature of 3 °C/min and a holding time of 2 h. The first portion was activated at 600 °C (denoted AC-S-600), the second and third portions were heated at 700 °C and 800 °C, then named AC-S-700 and AC-S-800, respectively.

### 2.2. Characterization Techniques for Activated Carbon (AC)

The thermogravimetric analysis (TGA) was executed on the AMC powder material and AC samples using the TGA-DSC analyzer (Jupiter STA449 F3 NTZSCH GmbH) Selb, Germany. The samples were heated in pure air at a flow rate of 10 cm^3^/min from room temperature to 1000 °C with a ramped temperature of 10 °C/min with a run time of 1 h and 40 min using calcinated silica pan as reference material [14,46,47]. The samples’ morphological characterization was carried out with the Field Emission Scanning Electron Microscope (FESEM) GeminiSEM 500 M/s Carl ZEISS-EDAX Z2 Analyzer AMETEK, Bangalore, India. The crystal structure of the AC powder samples was examined by X-Ray Diffraction (Riguku Smartlab Autosampler (RIGAKU Corp., Tokyo, Japan)) using a Cu kα radiation with the JCPDS-ICDD database. The surface functional groups of AC samples were analyzed by Fourier Transformation InfraRed (FTIR) spectroscopy (Bruker Optik GmbH Vertex 70, Ettlingen, Germany) and was scanned through a range of 600–4000 cm^−1^. The nitrogen adsorption/desorption isotherms were determined based on the automated adsorption instruments (11-2370 Gemini Miceomeritics, Atlanta, GA, USA). The AC samples were preheated at 90 °C (ramp 10 °C/min) and degassed at 300 °C in a vacuum with a holding time of 10 h. The nitrogen adsorption isothermal was measured over (P/Po) relative pressure and the BET surface area (S_BET_) was calculated by BET (Brunauer-Emmett-Teller (Atlanta, GA, USA) equation with adsorption data [14]. The micropore volume (*V*_mic_), microporous surface area (*S*_mic_) and external surface area (*S*_ext_) were obtained from the t-plot method [14]. The mesopore volume (*V*_mes_) was acquired by BJH (Barrett-Joyner-Halenda) method [14,42] and the total pore volume (*V*_tot_) estimated from the sum of mesoporous volume (*V*_mes_) and microporous volume (*V*_mic_). The mean pore radius (*r*_mp_) was determined from the total pore volume and S_BET_ with some assumption.

The surface chemistry of the AC samples was further examined by X-ray Photoelectron Spectroscopy (XPS) using spectra in the K-alpha Photoelectron spectrometer using omicron Nano Technology, London, UK. This was used to determine the chemical and elemental composition of the sample surfaces and for the identification of oxygen functional groups with their binding energy [18,31] and Labram Micro Raman Spectrometer (Horiba Jobin Yvon model, Minami-ku Kyoto Japan. Chemical titration Boehm method was used to further determine oxygen functional groups as follows; 200 mg of each AC sample was mixed in 25 mL of one of the four reactants of 0.1 M concentration [NaHCO_3_, Na_2_CO_3_, NaOH or HCl]. The mixtures were sonicated for 24 h, then filtered to remove the carbon. The excess of base and acid in solution was titrated with 0.1 M HCl solution of 0.1 M NaOH. The number of acidic and basic sites were calculated on the basis that NaOH neutralizes carboxylic, phenolic and lactonic groups, NaHCO_3_ neutralizes only carboxylic, Na_2_CO_3_ neutralizes carboxylic and lactonic and HCl neutralizes all the basic sites [48].

### 2.3. Electrochemical Preparation and Electrochemical Measurements

The working electrodes were fabricated with graphite foil as a current collector and AC as the active material. The active material constituted a mixture of AC, carbon black and Polyvinylidene Difluoride (PVDF) in the ratio of 8:1:1 by weight, respectively [49]. This was mixed with the N-methyl-2 pyrrolidone solvent to make a paste and coated on the graphite foil. The coated electrodes were dried in an oven at a temperature of 70 °C for 15 h. The electrochemical (EC) analysis was carried by BIO-LOGIC (BCS-805) work station using a three-electrode setup system with a saturated calomel electrode (SCE), coated material and a platinum sheet electrode as a reference, working and counter electrodes, respectively. The following techniques; (i) Cyclic Voltammetry (CV) with electrode potential of 1.0 V for 6 M KOH and 1.2 V for 1 M Na_2_SO_4_ at different scan rates (5, 10, 20, 50, 70, 100 mV/s). (ii) The Galvanostatic Charge-Discharge (GCD) performed at different current densities from 0.25, 0.5, 1.0, 1.5, to 2.0 A/g according to the total mass of the active electrodes in each device. (iii) Electrochemical Impedance Spectroscopy (EIS) was obtained from 10 kHz to 10 mHz at a voltage of 10 mV. (iv) Stability analysis of the SC was carried out by voltage holding/floating of the GCD after every 3 cycles for 10 h at the maximum voltage until 100 h, as described in References [18,50].

## 3. Results and Discussion

### 3.1. The Characterization of Activated Carbon AC

**Figure 1:**
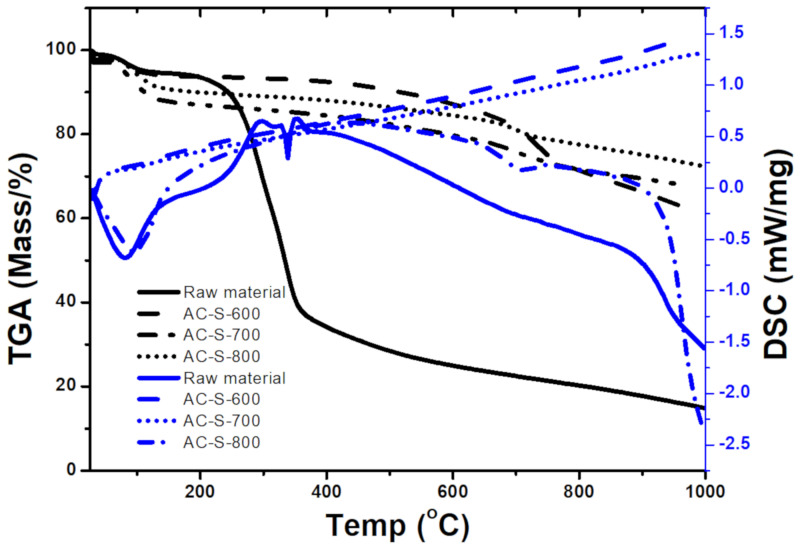
TGA-DSC analysis profile of African maize cobs (AMC) dried powder materials, AC-S-600, AC-S-700 and AC-S-800 activated carbons.

The thermal degradation and stability of the raw maize cobs (MC) and AC samples were performed with thermogravimetric analysis (TGA-DSC) at a temperature range of 28 to 1000 °C. Table S1 and Figure 1 represent the mass fraction loss in percentages at different temperature ranges. The dried powder of MC was analyzed to determine the required activation temperature. The MC sample shows two main stages of weight loss (Figure 1), with the first stage starting at approximately 28 °C up to 120 °C on the TGA graph. This was due to the desorption of water/moisture from the powder and showed an endothermic effect in the DSC heat flow profile that ended at 120 °C [47]. The second stage was observed between 200 °C and 350 °C (52.4%), which is due to the decomposition of organic compounds and groups such as carboxyl, lactonic, quinone and so forth, present at the surface of the material [14]. The highest mass loss at the second stage in MC up to 350 °C (Table S1) indicates that this can serve as the best carbonization temperature for the material. The AC materials’ activation was done starting from 600 °C where the MC material maintained a constant mass loss based on the TGA-DSC profile (Figure 1). The ACs profiles (Figure 1) depicts mass loss stages, between 70 °C and 120 °C for the first and between 650 °C and 770 °C for the second stage. The first stage corresponds to the water molecules’ desorption from the samples [47], which depicts an endothermic effect only in the AC-S-800 sample on the DSC profile. The second stage was due to the thermal degradation of organic compounds which corresponds to a weight loss range from 12–26% (Table S1) and may constitute loss of cellulose, hemicellulose and lignin compounds, which vaporize at that temperature range [51].

**Figure 2:**
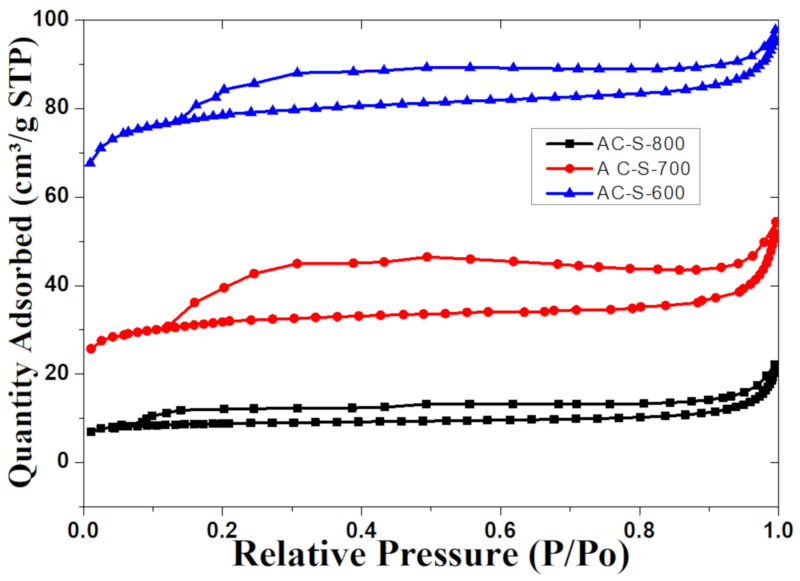
N_2_ adsorption-desorption isotherm at 77 K of Activated carbon samples.

**Figure 3:**
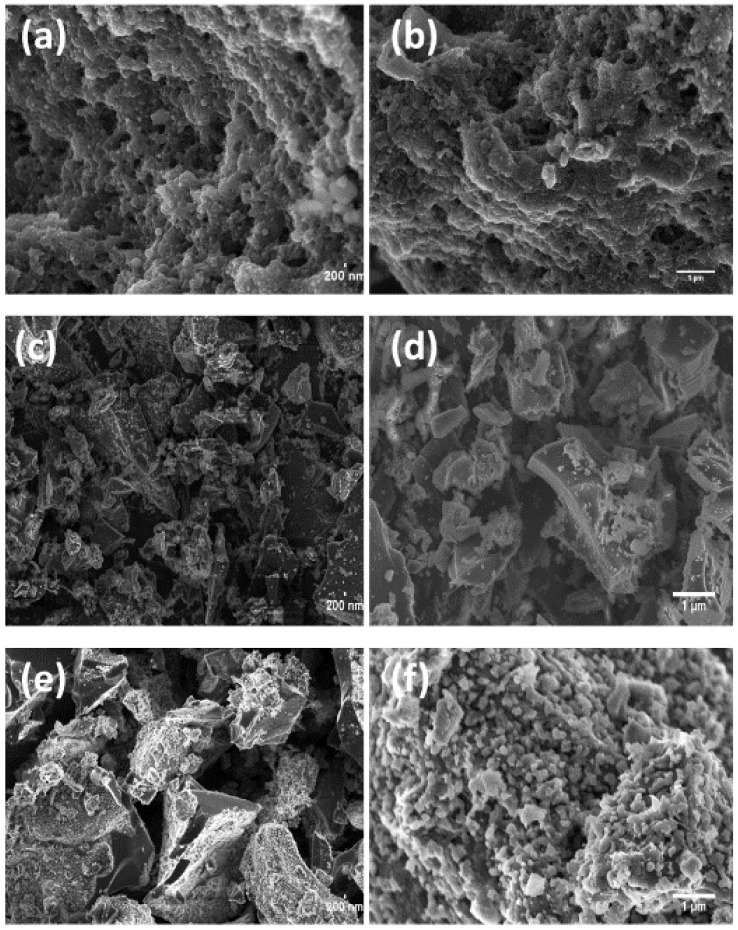
Field Emission Scanning Electron Microscopy (FESEM) micrographs for AC-S samples taken at two different magnifications AC-S-600 at (**a**) 200 nm, (**b**) 1 µm, AC-S-700 at (**c**) 200 nm, (**d**) 1 µm, AC-S-800 at (**e**) 200 nm, (**f**) 1 µm.

The DSC profile of AC-S-600 and AC-S-700 show a gradual increase in the heat flow (exothermic effect) throughout the samples, indicating that the samples are hydrophobic and gain more heat energy as temperature increases [35]. The AC-S-800 sample shows a different DSC profile with an endothermic effect at the first and second stages of the mass loss with a drastic effect at 950 °C until the end of the thermal analysis. This suggests that the AC-S-800 sample is highly microporous with high retention of gases and water molecules, indicating low pore volume and low surface area as confirmed by BET results (Figure 2) and the FESEM images (Figure 3). The mass loss increased with the increase in activation temperature in the order of AC-S-800 > AC-S-700 > AC-S-600 (Table S1). The decomposition generally increases for biomass with the elevation of temperature. The physical properties of the AC-S-600, AC-S-700, AC-S-800 AC samples was estimated by N_2_ adsorption-desorption as shown in Figure 2 and Table 1. The technique revealed the BET surface area (S_BET_) of 253.6 m^2^/g for AC-S-600, AC-S-700 with 105.23 m^2^/g and AC-S-800 with the lowest of 30.09 m^2^/g. The samples’ pore volume varies from 0.0312 to 0.1463 cm^3^/g with the increasing order of AC-S-800 < AC-S-700 < AC-S-600, as shown in Table 1.

The *S*_BET_ and pore volume decreased with an increase in the temperature used for each sample. The surface area decreases as the temperature increases and a similar trend was observed for the micropore volume, which decreases with increasing temperature. This indicates that H_2_SO_4_ acid-treated materials gave low physical properties and high chemical properties like oxidation and functionalization of the materials with an increase in temperature as detected by other techniques used in characterization (XPS, FTIR). This indicates that concentrated H_2_SO_4_ acid caused hydrolysis and oxidation reactions in the samples and the effect increased with the increase in the temperature. This effect leads to the blocking of the micropores and mesopores in the sample. When the temperature is increased beyond 200 °C, the H_2_SO_4_ acid-treated samples tend to generate polysulphates, which are highly corrosive and oxidizing functional groups, form by crosslinking polycondensation [52,53,54]. This leads to the formation of poly-form structures within the pores, hence closing the micropores and creating oxygen functional groups and trapping high oxygen concentration in the structure. The isothermal curves with hysteresis loops, as shown in Figure 2, depicted a type I and IV curves format of the isothermal classification by International Union of Pure and Applied Chemistry (IUPAC). The hysteresis of the curves has a sharp rise, which denotes the possible formation of mesopores from 0.4–0.9 P/Po, showing an important uptake at those relative pressures. The raise at >0.9 P/Po was caused by interparticle condensation of nitrogen [14]. The average pore distribution in all the samples implies that the materials can be used for storage applications. Using BJH analysis for pore size data, the average mean pore radius was estimated to be 0.577, 0.722, 1.04 nm with AC-S-600, AC-S-700 and AC-S-800, respectively using total pore volume and BET surface area. The pore size distribution for the samples in Figure S2 (in supporting document) shows a low range below 100 nm of the pores’ diameter, which favors the materials for supercapacitor application.

The FESEM micrographs of AC-S-600, AC-S-700 and AC-S-800 shown in Figure 3 depict the activated carbon materials’ physical morphology at different activation temperatures. AC-S-600 shows a honeycomb structure with different pore sizes and shapes, which is due to the removal of volatile compounds during carbonization. Figure 3c, presents a dense microstructure with smooth and fewer pores when compared to the others. The AC-S-800 (Figure 3f) sample revealed small pores in the microstructure, which may have been due to high oxidation properties of H_2_SO_4_ and the recombination of oxides from decarboxylation reaction with other functional groups [47,55,56]. The AC-S-600 sample exhibited removal of acidic group which vaporizes up to 650 °C. AC-S-700 sample (Figure 3d) exhibited slake likely structure particles indicating alkaline groups which vaporizes at 750 °C. AC-S-800 sample exhibited formation of poly forming surfaces of polysulphates at high temperatures.

**Figure 4:**
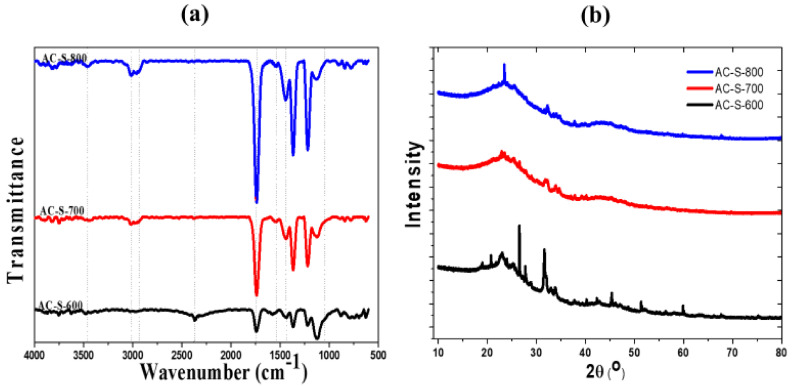
(**a**) Fourier transform infrared (FTIR) spectra and (**b**) X-ray diffraction (XRD) peaks for AC-S-600, AC-S-700 and AC-S-800 AC materials.

Similarly, the AC-S-800 sample depicted aggregated oxygen functional groups concentration on the surface as detected by the Boehm method. This was also confirmed by XPS (Figure 4) data values for oxygen percentage for surface atomic composition. Figure 3f at a 1 µm scale displayed a highly oxidized surface with 0.420 mmol/g of basic functional groups (Table S3). The basic groups are more in this sample since acidic groups decompose at around 650 °C, leaving the basic group decomposing between 700 °C and 980 °C [14]. Some of these basic groups are very beneficial for the supercapacitors’ performance, as indicated further in the electrochemical results.

The FTIR spectra of the AC-S-600, AC-S-700 and AC-S-800 activated carbon samples are presented in Figure 4a with functionality/group assignments shown in Table S2 (Supplementary Information). All three samples presented peaks at the same positions for each assignment for both regions. The fingerprint region from 1000–1500 cm^−1^ revealed the concentration of C–C, C–OH, C–O, C–N stretching and aromatic compounds with strong and medium transmittance. The peaks at 1080 and 1220 cm^−1^ corresponds to the stretching modes of the C–C and C–O bonds. Also, the diagnostic region revealed several groups in different positions as shown in Table S2 (in supporting document). These peak positions are in agreement with different activated carbon from other materials from the literature. Some of these materials include olive stones activated with phosphoric acid (H_3_PO_4_) [30], cotton stalk activated with H_3_PO_4_ [57], hydrochar activated with H_3_PO_4_ [55], maize cobs treated with ZnCl_2_ [58], fox nuts activated with H_3_PO_4_ [59], agricultural waste biomass activated with ZnCl_2_ and K_2_CO_3_ [51], cotton stalk with H_3_PO_4_ and ZnCl_2_ [47] and rice husks activated with NaOH [14].

The XRD spectra of the samples are presented in Figure 4b. The materials show two broad peaks at 2θ range of 23° to 30° and 40° to 47°, corresponding to the (002) and (110) crystal plane of graphite materials and it is reflected in all three samples. Generally, all the samples have shown broad peaks around 2θ of 24° and 44°, which indicates the typical peaks for amorphous carbon with non-crystalline material structures [42,52,60]. The AC-S-600 sample revealed a high-intensity peak at 2θ = 26.43° with a hexagonal crystal system of graphite with a *d*-spacing of 3.370 Å at (002) plane, indicating graphitization. The XRD pattern peaks of the samples correlated with perfect graphite crystal diffraction from (002) and (110) plane which are broad for all the samples as always found with amorphous and porous carbons. The broad peaks shown some spikes which indicate the presence of mixed phases. These spikes also suggest an unidentified well-crystalized inorganic impurity in the carbon samples, which may be due to the plant uptake from contaminated soils.

The surface chemistry of the functional groups in this study, which determines the acidity and basicity of the surface oxygen groups of the AC materials, were determined by the Boehm titration method and the results are shown in Table S3 (Supplementary Information). It is assumed that bases neutralize all oxygen groups that are more acidic in that NaHCO_3_ was used to deprotonate primarily carboxylic groups, Na_2_CO_3_ to neutralize lactonic groups and NaOH for deprotonation of phenolic groups. Also, HCl acid was used to protonate all the basic groups [48]. The total basicity if found to increases with an increase in the activation temperature because their decomposition is between 700 °C and 1000 °C [14] and the total acidity remains constant through for all samples (Table S3, Supplementary Information). These functional groups can enhance the performance of supercapacitors [14].

**Figure 5:**
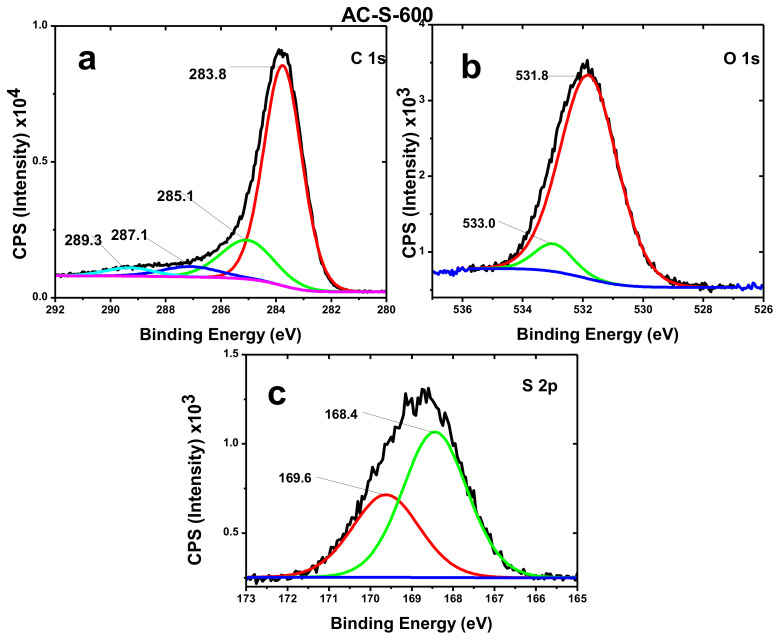
X-ray photoelectron spectroscopy (XPS) deconvoluted spectra of AC-S-600 activated carbon material (**a**) C 1s Carbon atom at 283.7 eV, (**b**) O 1s Oxygen atom at 531.7 eV, (**c**) S 2p Sulphur atom at 168.7 eV.

The XPS technique was further used to confirm the surface chemistry (the C1s, O1s and S2p core levels of atoms) by determining the material’s binding energy and the chemical state of the material’s surface [61]. This revealed the presence and interaction of different functional groups, which include C–C (C–H), C–O, C=O, O–C=O, C–S, among others, as depicted in Table S5 (Supplementary Information) [18,28,31,62]. The deconvoluted spectra (Figure 5) and Figure S1 (Supplementary Information) shows the C1s, O1s and S2p core level interaction in all three samples. From Table S4 (Supplementary Information), it was revealed that the carbon percentage is highest in AC-S-600 with 67.01% and oxygen with 22.59%. XPS is a surface sensitive technique which revealed a high percentage of oxygen on the thin surface of the materials. The trend of carbon composition is AC-S-600 > AC-S-700 > AC-S-800. In all the samples, there is approximately 2.6% sulfur detected in the samples. This is because the treatment was done with concentrated H_2_SO_4_ acid, which formed functional groups at the surfaces, as shown in Table S5 (Supplementary Information). There is some percentage composition of sodium and boron in the samples, which may be due to micro-nutrients uptake of the maize plants from the soils, they were cultivated. The concentration of these elements is approximately the same, implying that they came from the same source.

Table S4 (Supplementary Information) shows the convoluted spectra positions with their assignments and sample percentage content. This is in agreement with the Boehm analysis results in Table S3 (Supplementary Information) and FTIR results, indicating the oxidation and formation of functional groups at the surface of the materials. The spectra from the deconvoluted peaks of samples in Figure 5 for AC-S-600 and Figure S1 (for AC-S-700 and AC-S-800) indicate the formation of bonds with different binding energy as assigned in Table S5 (Supplementary Information). These have the same binding energy response for C1s, O1s and S2p for the three samples with no shift in the peaks as in Table S4 (Supplementary Information), indicating these are the main core atoms for the prepared AC materials. The convoluted C1s spectrum of AC-S-600 revealed peaks with the binding energy of 283.8 eV with the highest area indicating C-C and C=O carbon network. These binding energies and assignments are in agreement with the literature [18]. At the lower binding energy, the S2p spectrum was revealed in all the samples forming polysulphates groups of O–S=O, O–C–S, which arise due to the chemical activation H_2_SO_4_ [63]. This is explained based on the fact that sulfur atoms may be introduced in the carbon matrix at reduced states that are further stabilized by oxidation, leading to stable S-functional groups. The increase in oxygen content with an increase in temperature may be due to the formation of polysulphates, which block the pores and trap the oxygen. This may reduce the conductivity but increases the functional groups on the material surface, which increases the functionality of the faradaic reactions.

**Figure 6:**
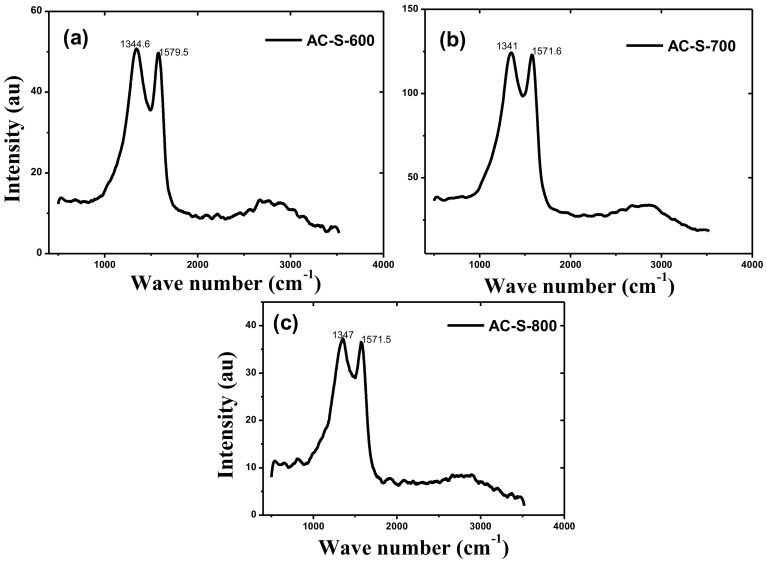
Raman spectra for (**a**) AC-S-600, (**b**) AC-S-700 and (**c**) AC-S-800 activated carbon materials.

The Raman spectra of the three AC-s materials are shown in Figure 6. The samples show peaks at 1355 cm^−1^ for the D-band and 1597 cm^−1^ for G-band. These peaks indicate the formation of disordered carbon and graphitic carbon for D-band and G-band, respectively [64]. The intensity ratio of *I*_D_/*I*_G_ indicates the disordered carbon, which is 1.02, 1.01 and 1.02 for AC-S-600, AC-S-700 and AC-S-800, respectively. These values of *I*_D_/*I*_G_ present a good degree of graphitization of the produced materials with predominant amorphous carbon domain.

### 3.2. The Electrochemical Characterization of Activated Carbon

**Figure 7:**
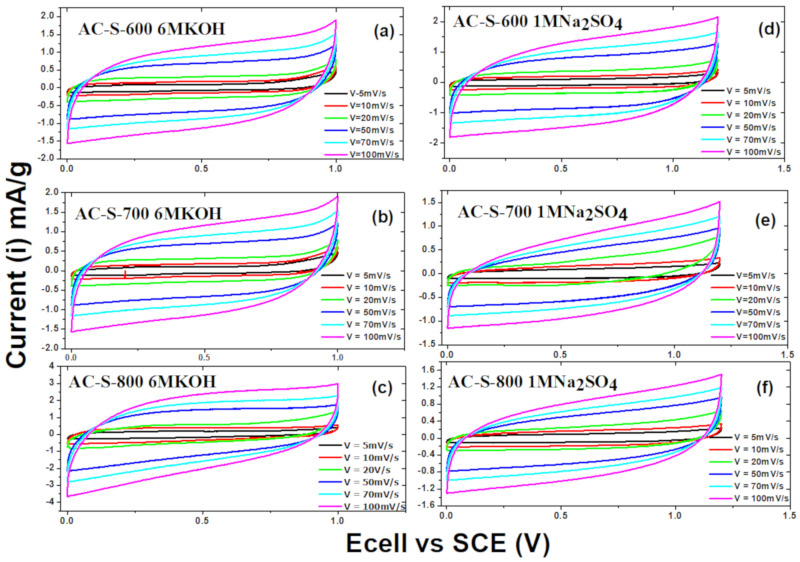
Cyclic Voltammetry (CV) curves of AC-S-600, AC-S-700 and AC-S-800 electrode materials at different scan rates with 6 M KOH (**a**–**c**) and 1 M Na_2_SO_4_ (**d**–**f**) as electrolytes.

The characterization of the materials as electrodes for supercapacitor application was evaluated by cyclic voltammetry (CV) and galvanostatic charge discharge (CD) curves. By using three electrodes system the electrodes of the supercapacitor, the calculation was done based on the following equations [3,65,66,67];

(1) $$C_{s}=\;\frac{Idt}{m_{el}dV}$$

where; *C*_s_ is the specific capacitance (F/g), *I* the discharge current (A), *m*_el_ the total mass of active materials (g), dV the change in discharge voltage (*V*) and dt the change in discharge time (s). The results of the setups made from the AC (AC-S-600, AC-S-700 and AC-S-800) samples were analyzed with two different electrolytes, namely: potassium hydroxide (6 M KOH) and sodium sulphate (1 M Na_2_SO_4_). In the assessment of the cyclic voltammetry, the voltammograms from the two electrolytes were obtained using five different scan rates of 5, 10, 20, 50, 70 and 100 mV/s at a voltage window of 0–1.0 V for 6 M KOH and 0–1.2 V for 1 M Na_2_SO_4_. All three-electrode materials display rectangular and semi-rectangular shapes at different scan rates and with different voltage windows, as shown in Figure 7. These characteristics can be assigned to the electrical double layer supercapacitor (EDLS) behaviors for all three electrodes. This means there was a low electrolyte diffusional restriction with the materials [18]. The electrode maintains its rectangular shape with an increasing scan rate, indicating excellent capacitive nature with a rapid response to the electrode materials’ electrochemical interaction, as in Figure 7. This may be due to the ions’ easy transport through the pores in the formation of the material under study [68] and further indicates small resistance in the samples as electrode materials [69]. There is some exponential distortion in some quasi-rectangular shapes with higher scan rates due to high polarization that indicates that there was some overcharging of the electrodes. This resulted in a parasitic side reaction that may have occurred with the electrode materials or the electrolyte [70]. Figure S3 (Supplementary Information) show some small humps and exponential character, which may be attributed to the acidic groups (Table S3, Supplementary Information), there was no pseudo-faradaic interaction reaction with the different surface functional groups on the surfaces of the materials. The rectangular features maybe due to the controlled working range which does not all scanning in the faradaic range. Also, the electrode material exhibited a good wettability property (Table S5, Supplementary Information) [14]. The slight difference in the shapes is due to the conductivity differences of the electrolytes used [61] and the long contact time at high potential during stability testing.

**Figure 8:**
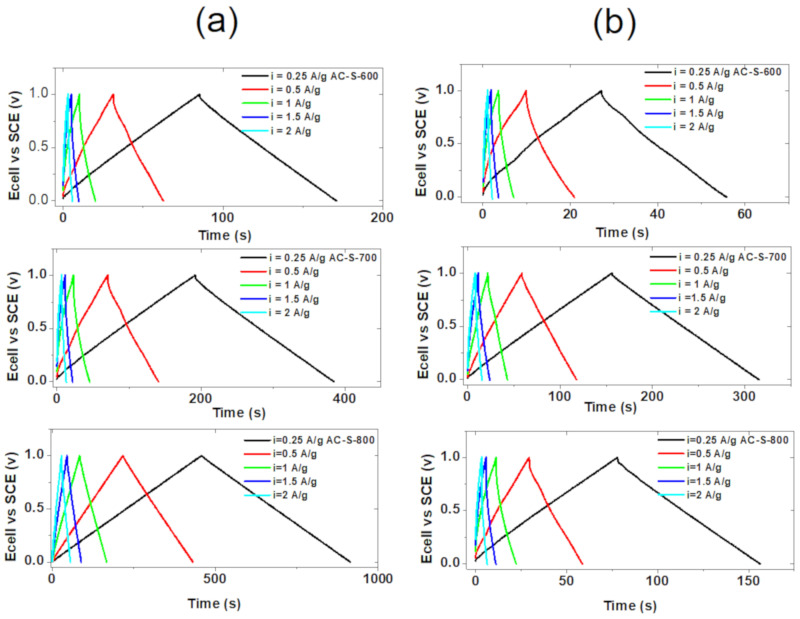
Galvanostatic charge-discharge (GCD) curves for AC-S-600, AC-S-700 and AC-S-800 electrodes materials at different current densities with (**a**) 6 M KOH and (**b**) 1 M Na_2_SO_4_ as electrolytes.

The galvanostatic charge/discharge (GCD) of the electrodes was studied using the chronopotentiometry technique. The GCD from the different electrode materials is presented in Figure 8 at varying specific current and different potential windows for both electrolytes, as mentioned earlier. The current density was varied from 0.25−2.0 A/g for all three electrodes. Triangular symmetric GCD was observed for all samples, as shown in Figure 8. This symmetric behavior is a measure of an ideal electrical double-layer capacitor (EDLCs). The deviation from the ideal EDLC could be due to (i) current leakage limited by the poor assembly of the device to be tested, (ii) lack of adequate electronic conductivity arrangement or high series resistance, (iii) side reaction which occurs within the electrolyte or with the active electrode material [70]. As the current density increases, there is a slight distortion to the triangular shape using KOH electrolyte (Figure S4). This may be due to resistance in the system or limited diffusion of potassium ions (K^+^) into the active material’s pores at high current [14]. The molar conductivity of a strong electrolyte depends slightly on the concentration but a strong electrolyte is fully ionized in solution with increased ion-ion interaction. This makes the ions to migrate independently causing ion mobility following the law of independent migration. KOH electrolyte has low limiting molar conductivity (25.36 mS m^2^ mol^−1^) compared to Na_2_SO_4_ electrolyte (26.02 mS m^2^ mol^−1^). Hence, KOH has high mobility compared to Na_2_SO_4_ electrolyte giving higher electrochemical performance as showed in the current results [69]. The Specific capacitance for KOH electrolyte was decreasing linearly compared to that Na_2_SO_4_ electrolyte, as shown in Figure S4 (Supplementary Information).

The two electrolytes were used to compare the activities of the materials in the basic and neutral environment. The data in Figure 8 show the results of the materials’ activities during the electrochemical (EC) analysis. The electrodes from the AC-S-600 in 6 M KOH electrolyte gave a specific capacitance of 85.25 F/g 0.25 A/g while the same material in 1 M Na_2_SO_4_ electrolyte gave a specific capacitance 28.43 F/g at the same specific current. The AC-S-700 AC electrode in 6 M KOH gave 172.08 F/g at 0.25 A/g, which is higher than AC-S-600 with the same electrolyte. The electrode fabricated from the AC-S-700 in 1 M Na_2_SO_4_ had a specific capacitance of 159.02 F/g at 0.25 A/g. Furthermore, the AC-S-800 electrodes in 6 M KOH electrolyte gave the highest specific capacitance of 456.4 F/g and 78.5 F/g at 0.25 A/g in 1 M Na_2_SO_4_ electrolyte. The obtained specific capacitance is higher compared to those in the literature, as shown in Table 2. Earlier reports show that the hydrated ion size (3.31 Å for K^+^ and 3.58 Å for Na^+^) and ionic conductivity (73.5 S cm^2^ mol^−1^ for K^+^ compared to 50.11 S cm^2^ mol^−1^ for Na^+^) play a crucial role in the electrochemical performance of carbon electrode materials [71]. Similarly, the ionic radius of the hydrated negatively charged anions contribute to the EDLC behavior via electrosorption and the sizes are in the following order OH^−^ (3.00 Å) < NO_3_^−^ (3.35 Å) < SO_4_^2−^ (5.33 Å) [71]. Hence the alkaline electrolyte is expected to give the best electrochemical performance, taking into account the micro and mesoporous texture of the carbon electrode that could easily accommodate the smaller size of K^+^ and the electrosorption of the negative charge anions (OH^−^), coupled with its better conductivity and ionic mobility [72]. The acid-activation introduced sulphur into the electrode material. Sulphur is an active element for energy storage material with a theoretical capacity of 1672 mAh g^−1^ and theoretical specific energy of 2600 Wh kg^−1^. This can improve the reaction completion for electrolyte ion to form sulphide ions. Also, sulphur forms multiple strong bonds with carbon surfaces because of its low electronegativity (2.58). This is capable of forming more redox-active sites, which can enhance the performance of the electrode. Sulphur also improves the stability, electrical and surface properties and the electrode materials’ wettability, hence exhibiting high retention and high charge/discharge cycle stability [3]. The functional groups have specific effects. Quinone-hydroquinone are organic redox systems with electrochemical behavior associated with electron-proton transfer equilibrium. The undergo reversible two-electron redox which vary with pH. The potential of the EC performance depends on stability of species reduced, electrolyte used and the presence of another functional group play a crucial role. The EC performance of carbonyl functional groups depends solely on the charge state of carbon and oxygen atoms of the groups which balances with the counter ion in the electrolyte salt for reaction.

**Figure 9:**
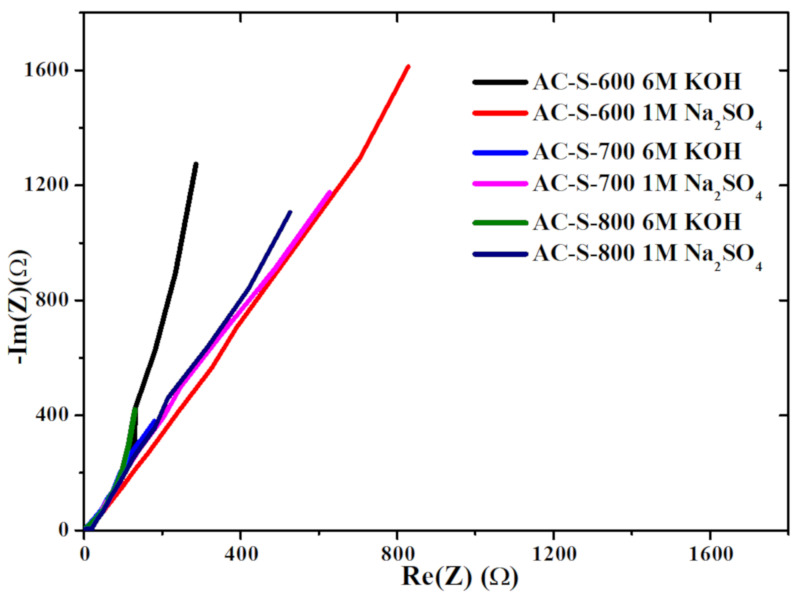
Electrochemical Impedance Spectroscopy (EIS) Nyquist plot for AC-S-600, AC-S-700 and AC-S-800 for two different electrolytes (6 M KOH and 1 M Na_2_SO_4_) at the potential of 10 mV.

Nyquist plots of the electrode materials are shown in Figure 9 for the different electrolytes. For the electrode materials made from for AC-S-800 material, the impedance data was collected at different potentials to ascertain the origin of the charge transfer resistance (*R*_CT_) and interfacial resistance, as shown in Figure S5 (Supplementary Information). The potential was varied from 5–20 mV with both electrolytes as revealed in Figure S5a,b, Supplementary Information. From Figure 9, the Nyquist plots for all the materials depicted the same shapes, different sizes of the semicircle and Equivalent Series Resistance (ESR) at high-frequency regimes and almost the same behaviors at low-frequency regimes. This may be attributed to the presence of functional groups or other impurities (dopants) on the surface of the material and the current collector [70,73]. These may have caused less charge transfer resistance contributing to a less doom shaped arch curve (Figure S7, Supplementary Information). The ESR of the AC-S-600 electrode is approximated at ~2.5 Ω for both electrolytes (Figure S7). The ESR for AC-S-700 and AC-S-800 is estimated between 1–2.5 Ω hence good performance. These values take into account the different factors, which include the internal resistance components of the cells and electrolyte. The AC-S-800 material with 6 M KOH revealed no semicircle at high frequency (Figure S7) with a 45° line from ESR, indicating charge storage of a typical AC with functional groups on the surface porous with no charge transfer [70].

In the EIS data analysis, the study revealed conductive material properties for electrode application by showing low ESR values, indicating low internal resistance within the material, current collector, electrolyte and the cell components [73]. In Figure S5a,b, Supplementary Information, the potential was varied to examine the effect of charge transfer resistance (*R*_CT_) or change in ESR. The response did not vary and never changed the impedance spectra at a lower frequency. This confirmed no charge transfer resistance, only the interfacial impedance, which occurs at constant *R*_CT_ [70].

The stability of the cells was tested after CV, GCD and EIS measurement by the floating test method, as described in References [50,69,73,74,75], which is called voltage holding or voltage floating or aging of the cell. In the current study, the stability was set to have three GCD then at the fourth charge maximum, the voltage was held for 10 h before the next 3 GCD and another holding. This was carried out for 100 h of holding. The capacitance at every set was calculated using the second GCD cycle using 0.25 A/g at a maximum potential window of 1.0 V for 6 M KOH electrolyte and 1.2 V for 1 M Na_2_SO_4_ electrolyte. Figure S6b,c, Supplementary Information, shows the stability plots with capacitance as a function of floating/holding time. The capacitance for AC-S-600 with 6 M KOH showed some stability for the first 60 h then dropped for 20 h before stabilizing again (Figure S6b). This was almost the same case for the same material with the second electrolyte by dropping after 70 h and never stabilized (Figure S6c).

The AC-S-700 and AC-S-800 materials revealed a more stable holding with both electrolytes and a little raise-drop with AC-S-700 with 6 M KOH at the beginning. The experiment was set to analyze the degradation effect of the active materials and the electrolyte by holding the cell at its maximum voltage of operation. In Figure S6c, Supplementary Information, any raise in the capacitance after 40 h may be due to increased pore interaction as the electrolyte intercalates through the porous material. The drop in the capacitance from the lowest did not reach 20% since the degradation is considered between 20% and 30% losses of its capacitance [50]. After 100 h, the cells still showed good performance demonstrating negligible degradation [76].

The self-discharge of the cell was performed on the best cell with AC-S-800 using 6 M KOH. The cell was charged to a maximum of 1.0 V and held for 5 min before it was left at an open system of circuit potential to go through a self-discharge for hours, as shown in Figure S6a (Supplementary Information). The cell took more than 12 h to reach 30% loss and was examined for more than 16 h and never reached half-maximum voltage loss compared to results in the literature [77]. The self-discharge mechanism is comparable with the Gibbs energy difference between charge and discharge states [69], which can explain the self-discharge phenomena. The voltage drop in 4 h is associated with the electrolyte instability, which causes some degradation by the generation of gases since the electrolyte is near its ionic dissociation [78,79]. This device exhibited stable electrochemical stability, which can be used for standby applications.

**Table 2:** Performance of supercapacitors with different electrode materials from different biomass sources chemically treated with different impregnation from literature in comparison with this current study.

Electrode Material	Chemical Activation	Electrolyte Used	Current Density (A/g)	Capacitance (F/g)	Ref.
Corn Cob	KOH	6 M KOH	-	309.81	[42]
Banana Stem	KOH	6 M HOH	-	479.23	[42]
Olive Residues	KOH/R	1 M H_2_SO_4_	0.25	224	[18]
Olive Residues	KOH/R	1 M Na_2_SO_4_	0.25	193	[18]
Nanoporous Carbon	K_2_CO_3_	2.5 M KNO_3_	0.5	140	[69]
Rice Husks	NaOH	0.5 M K_2_SO_4_	1.0	198.4	[14]
Coniferous-Pine-Biomass	KOH	1 M Na_2_SO_4_	0.1	90	[49]
Pinecone	KOH	3.5 M KNO_3_	0.5	300	[80]
Hemp Straw	KOH	6 M KOH	1.0	244	[60]
Pinewood	H_3_PO_4_/KOH	6 M KOH	1.0	366	[52]
AMC	H_2_SO_4_	6 M KOH	0.25	456.4	This Study
AMC	H_2_SO_4_	1 M Na_2_SO_4_	0.25	159.02	This Study

## 4. Conclusions

This research studied the value addition to corn cobs biomass towards AC materials for supercapacitors. The process of converting AMC biomass into AC material was achieved successfully by carbonization (functionalization) of the precursor with concentrated Sulphuric acid and was activated in three (3) batches at different temperatures 600, 700 and 800 °C using a furnace in a nitrogen atmosphere. Acid functionalization indicated the addition of oxygen functional groups on the surface of the structure. The activated carbon materials were characterized and examined for physical and chemical properties, including as electrode materials for supercapacitor applications. The AC materials revealed low porous structures with *S*_BET_ between 30 m^2^/g and 254 m^2^/g, the total pore volume of 0.0312–0.1463 cm^3^/g and a mean pore radius of 0.577–1.04 nm. The materials were highly functionalized with oxygen groups on the surface, exhibiting a total acidity between 0.440 mmol/g and 0.464 mmol/g, total basicity of 0.09–0.420 mmol/g and oxygen composition of 22–32% for surface atomic composition. This greatly improved the GCD’s performance giving specific capacitance of 456.4 F/g at 0.25 A/g with 6 M KOH electrolyte and 159.02 F/g at 0.25 A/g with 1 M Na_2_SO_4_ electrolyte. Our results demonstrate corncobs derived activated carbons are promising electrode material for the construction of electrochemical supercapacitors.

## Figures and Tables

**Table 1:** Physical properties of AC samples from N_2_ adsorption at 350 °C.

Samples	*S*_BET_ (m^2^/g)	*S*_mic_ (m^2^/g)	*S*_mic_/*S*_BET_ (%)	*S*_ext_ (m^2^/g)	*V*_mic_ (cm^3^/g)	*V*_mes_ (cm^3^/g)	*V*_tot_ (cm^3^/g)	*V*_mic_/*V*_tot_ (%)	*r*_mp_ (nm)
AC-S-600	253.60	204.33	80.6	49.27	0.0998	0.0465	0.1463	68.2	0.577
AC-S-700	105.23	68.61	65.2	36.62	0.0330	0.0430	0.0760	43.4	0.722
AC-S-800	30.09	21.17	70.4	8.92	0.0098	0.0214	0.0312	31.4	1.041

## Supplementary Materials

The following are available online at https://www.mdpi.com/1996-1944/13/23/5412/s1, Table S1: The residual mass of raw material powder of maize corn cobs, AC-S-600, AC-S-700, and AC-S-800 activated carbon materials at the different temperature range, Table S2: FTIR peaks assignment for functionality/groups on AC surfaces for AC-S-600, AC-S-700, and AC-S-800 materials, Table S3: Boehm Acidic and Basic surface characterization of the activated carbon materials, Table S4: XPS mass surface concentration of activated carbon material’s composition (%), binding energy position and their Full Width at Half Maximum intensity (FWHM), Table S5: XPS spectra of AC-S-600, AC-S-700, and AC-S-800 activated materials, Binding Energy, Functional group assignment and their relative chemical bonding contents, Figure S1: XPS deconvoluted spectra of AC-S-800 and AC-S-700 activated carbon material, Figure S2: Pole size distribution for the activated carbon material samples, Figure S3: Combined CV curves of AC-S-Activated carbon samples at scan rates 5, 10 and 20 mV/s with 6M KOH and 1 M Na_2_SO_4_ electrolyte, Figure S4: specific Capacitance (F/g) with current density (A/g) curves plot for AC-S-600, AC-S-700, and AC-S-800 for two different electrolytes (6M KOH and 1 M Na_2_SO_4_), Figure S5: EIS Nyquist plots for AC-S-800 electrode material at different potentials using different electrolytes (a) 6 M KOH at 5 mV, 10 mV and 20 mV, (b)1 M Na_2_SO_4_ at 10 mV and 20 mV, Figure S6: (a) The self-discharge of the device with the highest capacitance assembled with AC-S-800 with 6M KOH after charging to 1.0 V, (b) Voltage holding stability capacitances of AC-S-600, AC-S-700, and AC-S-800 with 6 M KOH electrolyte at the current density of 1.0.A/g for 100 h, (c) stability of AC-S-600, AC-S-700, and AC-S-800 with 1 M Na_2_SO_4_ electrolyte at the current density of 1.0 A/g with 3 charges/discharge cycles and charge hold for 10 hours then repeated for 100 h, Figure S7: Electrochemical Impedance Spectroscopy (EIS) Nyquist plot for AC-S-600, AC-S-700, and AC-S-800 for two different electrolytes (6 M KOH and 1 M Na_2_SO_4_) at the potential of 10 mV at low frequency.

## Data Accessibility

All data generated or evaluated during this research study is included in this paper.

## References

[1] Hall P.J., Mirzaeian M., Fletcher S.I., Sillars F.B., Rennie A.J.R., Shitta-Bey G.O., Wilson G., Cruden A., Carter R. (2010). Energy storage in electrochemical capacitors: Designing functional materials to improve performance. Energy Environ. Sci..

[2] Zhang L.L., Zhou R., Zhao X.S. (2010). Graphene-based materials as supercapacitor electrodes. J. Mater. Chem..

[3] Tarimo D.J., Oyedotun K.O., Mirghni A.A., Sylla N.F., Manyala N. (2020). High energy and excellent stability asymmetric supercapacitor derived from sulphur-reduced graphene oxide/manganese dioxide composite and activated carbon from peanut shell. Electrochim. Acta.

[4] Nankya R., Opar D.O., Kim M.-J., Paek S.-M., Jung H. (2020). Synergetic effect of nitrogen and sulfur co-doping in mesoporous graphene for enhanced energy storage properties in supercapacitors and lithium-ion batteries. J. Solid State Chem..

[5] Dörfler S., Felhősi I., Marek T., Thieme S., Althues H., Nyikos L., Kaskel S. (2013). High power supercap electrodes based on vertical aligned carbon nanotubes on aluminum. J. Power Sources.

[6] Gao F., Qin S.-H., Zang Y.-H., Gu J.-F., Qu J. (2020). Highly efficient formation of Mn3O4-graphene oxide hybrid aerogels for use as the cathode material of high performance lithium ion batteries. New Carbon Mater..

[7] Zhai Y., Dou Y., Zhao D., Fulvio P.F., Mayes R.T., Dai S. (2011). Carbon Materials for Chemical Capacitive Energy Storage. Adv. Mater..

[8] Yeon S.-H., Kim D.-H., Lee S.-H., Nam S.-S., Park S.-K., So J.Y., Shin K.-H., Jin C.-S., Park Y., Kang Y.C. (2017). High microporosity of carbide-derived carbon prepared from a vacuum-treated precursor for energy storage devices. Carbon.

[9] Zhu Y., Murali S., Stoller M.D., Ganesh K.J., Cai W., Ferreira P.J., Pirkle A., Wallace R.M., Cychosz K.A., Thommes M. (2011). Carbon-Based Supercapacitors Produced by Activation of Graphene. Science.

[10] Lee Y.J., Kim G.-P., Bang Y., Yi J., Gil Seo J., Song I.K. (2014). Activated carbon aerogel containing graphene as electrode material for supercapacitor. Mater. Res. Bull..

[11] Vlad A., Singh N., Melinte S., Gohy J.-F., Ajayan P.M. (2016). Carbon Redox-Polymer-Gel Hybrid Supercapacitors. Sci. Rep..

[12] Das D., Samal D.P., Meikap B. (2015). Preparation of Activated Carbon from Green Coconut Shell and its Characterization. J. Biosens. Bioelectron..

[13] Lekakou C., Moudam O., Markoulidis F., Andrews T., Watts J.F., Reed G.T. (2011). Carbon-Based Fibrous EDLC Capacitors and Supercapacitors. J. Nanotechnol..

[14] Le Van K., Thi T.T.L. (2014). Activated carbon derived from rice husk by NaOH activation and its application in supercapacitor. Prog. Nat. Sci..

[15] Dhillon R., Von Wuehlisch G. (2013). Mitigation of global warming through renewable biomass. Biomass Bioenergy.

[16] Liu N., Huo K., McDowell M.T., Zhao J., Cui Y. (2013). Rice husks as a sustainable source of nanostructured silicon for high performance Li-ion battery anodes. Sci. Rep..

[17] Zhang L., Wang Y., Peng B., Yu W., Wang H., Wang T., Deng B., Chai L., Zhang K., Wang J. (2014). Preparation of a macroscopic, robust carbon-fiber monolith from filamentous fungi and its application in Li–S batteries. Green Chem..

[18] Elmouwahidi A., Bailón-García E., Pérez-Cadenas A.F., Maldonado-Hódar F.J., Carrasco-Marín F. (2017). Activated carbons from KOH and H3PO4-activation of olive residues and its application as supercapacitor electrodes. Electrochim. Acta.

[19] Elmouwahidi A., Zapata-Benabithe Z., Carrasco-Marín F., Moreno-Castilla C. (2012). Activated carbons from KOH-activation of argan (Arganiaspinosa) seed shells as supercapacitor electrodes. Bioresour. Technol..

[20] Wei T., Zhang Q., Wei X., Gao Y., Li H. (2016). A Facile and Low-Cost Route to Heteroatom Doped Porous Carbon Derived from Broussonetia Papyrifera Bark with Excellent Supercapacitance and CO_2_ Capture Performance. Sci. Rep..

[21] Hou J., Cao C., Ma X., Idrees F., Xu B., Hao X., Lin W. (2015). From Rice Bran to High Energy Density Supercapacitors: A New Route to Control Porous Structure of 3D Carbon. Sci. Rep..

[22] Zhu Z., Jiang H., Guo S., Cheng Q., Hu Y., Li C. (2015). Dual Tuning of Biomass-Derived Hierarchical Carbon Nanostructures for Supercapacitors: The Role of Balanced Meso/Microporosity and Graphene. Sci. Rep..

[23] Wei L., Sevilla M., Fuertes A.B., Mokaya R., Yushin G. (2011). Hydrothermal Carbonization of Abundant Renewable Natural Organic Chemicals for High-Performance Supercapacitor Electrodes. Adv. Energy Mater..

[24] Biswal M., Banerjee A., Deo M., Ogale S. (2013). From dead leaves to high energy density supercapacitors. Energy Environ. Sci..

[25] Mi J., Wang X.-R., Fan R.-J., Qu W.-H., Li W.-C. (2012). Coconut-Shell-Based Porous Carbons with a Tunable Micro/Mesopore Ratio for High-Performance Supercapacitors. Energy Fuels.

[26] Tian W., Gao Q., Tan Y., Yang K., Zhu L., Yang C., Zhang H. (2015). Bio-inspired beehive-like hierarchical nanoporous carbon derived from bamboo-based industrial by-product as a high performance supercapacitor electrode material. J. Mater. Chem. A.

[27] Chen X., Zhang J., Zhang B., Dong S., Guo X., Shanmu D., Fei B. (2017). A novel hierarchical porous nitrogen-doped carbon derived from bamboo shoot for high performance supercapacitor. Sci. Rep..

[28] Barroso-Bogeat A., Alexandre-Franco M., Fernández-González C., Gómez-Serrano V. (2019). Activated carbon surface chemistry: Changes upon impregnation with Al(III), Fe(III) and Zn(II)-metal oxide catalyst precursors from NO3-aqueous solutions. Arab. J. Chem..

[29] Szczypta A.F., Rabiej S., Szparaga G., Pabjanczyk-Wlazlo E., Król P., Brzezinska M., Blazewicz S., Boguń M. (2015). The structure and properties of the carbon non-wovens modified with bioactive nanoceramics for medical applications. Mater. Sci. Eng. C.

[30] Yakout S., El-Deen G.S. (2016). Characterization of activated carbon prepared by phosphoric acid activation of olive stones. Arab. J. Chem..

[31] Shafeeyan M.S., Daud W.M.A.W., Houshmand A., Shamiri A. (2010). A review on surface modification of activated carbon for carbon dioxide adsorption. J. Anal. Appl. Pyrolysis.

[32] Tang H., Gao P., Liu X., Zhu H., Bao Z. (2014). Bio-derived calcite as a sustainable source for graphene as high-performance electrode material for energy storage. J. Mater. Chem. A.

[33] Xu J., Gao Q., Zhang Y., Tan Y., Tian W., Zhu L., Jiang L. (2014). Preparing two-dimensional microporous carbon from Pistachio nutshell with high areal capacitance as supercapacitor materials. Sci. Rep..

[34] Lee M., Kim G.-P., Song H.D., Park S., Yi J. (2014). Preparation of energy storage material derived from a used cigarette filter for a supercapacitor electrode. Nanotechnology.

[35] Li X., Xing W., Zhuo S., Zhou J., Li F., Qiao S.-Z., Lu G.-Q. (2011). Preparation of capacitor’s electrode from sunflower seed shell. Bioresour. Technol..

[36] Kumagai S., Sato M., Tashima D. (2013). Electrical double-layer capacitance of micro- and mesoporous activated carbon prepared from rice husk and beet sugar. Electrochim. Acta.

[37] Shahbandeh M. Corn Production Worldwide 2019/2020, by Country. Statista. https://www.statista.com/statistics/254292/global-corn-production-by-country/.

[38] Ding J., Wang H., Li Z., Cui K., Karpuzov D., Tan X., Kohandehghan A., Mitlin D. (2015). Peanut shell hybrid sodium ion capacitor with extreme energy–power rivals lithium ion capacitors. Energy Environ. Sci..

[39] Wang H., Xu Z., Kohandehghan A., Li Z., Cui K., Tan X., Stephenson T.J., King’Ondu C.K., Holt C.M.B., Olsen B.C. (2013). Interconnected Carbon Nanosheets Derived from Hemp for Ultrafast Supercapacitors with High Energy. ACS Nano.

[40] Raymundo-Piñero E., Cadek M., Béguin F. (2009). Tuning Carbon Materials for Supercapacitors by Direct Pyrolysis of Seaweeds. Adv. Funct. Mater..

[41] Qian W., Sun F., Xu Y., Qiu L., Liu C., Wang S., Yan F. (2014). Human hair-derived carbon flakes for electrochemical supercapacitors. Energy Environ. Sci..

[42] Ghosh S., Santhosh R., Jeniffer S., Raghavan V., Jacob G., Nanaji K., Kollu P., Jeong S.K., Grace A.N. (2019). Natural biomass derived hard carbon and activated carbons as electrochemical supercapacitor electrodes. Sci. Rep..

[43] Yang S., Zhang K. (2018). Converting Corncob to Activated Porous Carbon for Supercapacitor Application. Nanomaterials.

[44] Cui L., Yang Y., Cheng C., Xu L., Li Y., Jia M., Dun X., Jin X. (2019). Corn Cob Lignin-based Porous Carbon Modified Reduced Graphene Oxide Film for Flexible Supercapacitor Electrode. J. Wood Chem. Technol..

[45] Wei Q.-L., Chen Z.-M., Wang X.-F., Yang X., Wang Z.-C. (2018). A two-step method for the preparation of high performance corncob-based activated carbons as supercapacitor electrodes using ammonium chloride as a pore forming additive. New Carbon Mater..

[46] Lim W., Srinivasakannan C., Balasubramanian N. (2010). Activation of palm shells by phosphoric acid impregnation for high yielding activated carbon. J. Anal. Appl. Pyrolysis.

[47] Hu L., Peng Y., Wu F., Peng S., Li J., Liu Z. (2017). Tubular activated carbons made from cotton stalk for dynamic adsorption of airborne toluene. J. Taiwan Inst. Chem. Eng..

[48] Schönherr J., Buchheim J.R., Scholz P., Adelhelm P. (2018). Boehm Titration Revisited (Part I): Practical Aspects for Achieving a High Precision in Quantifying Oxygen-Containing Surface Groups on Carbon Materials. J. Carbon Res..

[49] Manyala N., Bello A., Barzegar F., Khaleed A.A., Momodu D.Y., Dangbegnon J.K. (2016). Coniferous pine biomass: A novel insight into sustainable carbon materials for supercapacitors electrode. Mater. Chem. Phys..

[50] Weingarth D., Foelskeschmitz A., Kötz R. (2013). Cycle versus voltage hold—Which is the better stability test for electrochemical double layer capacitors?. J. Power Sources.

[51] Köseoğlu E., Akmil-Başar C. (2015). Preparation, structural evaluation and adsorptive properties of activated carbon from agricultural waste biomass. Adv. Powder Technol..

[52] Li J., Han K., Qi J., Teng Z., Li M., Wang M. (2019). Biomass-derived 3D hierarchical porous carbon by two-step activation method for supercapacitor. J. Mater. Sci. Mater. Electron..

[53] Liou T.-H. (2010). Development of mesoporous structure and high adsorption capacity of biomass-based activated carbon by phosphoric acid and zinc chloride activation. Chem. Eng. J..

[54] Chen C., Xie D.H., Chen X.Y., Zhang Z.J. (2013). High performance porous carbon through hard–soft dual templates for supercapacitor electrodes. J. Mater. Chem. A.

[55] Chen J., Zhang L., Yang G., Wang Q., Li R., Lucia L.A. (2017). Preparation and Characterization of Activated Carbon from Hydrochar by Phosphoric Acid Activation and its Adsorption Performance in Prehydrolysis Liquor. Bioresources.

[56] Zhang S., Pan N. (2015). Supercapacitors Performance Evaluation. Adv. Energy Mater..

[57] Nahil M.A., Williams P.T. (2012). Pore characteristics of activated carbons from the phosphoric acid chemical activation of cotton stalks. Biomass Bioenergy.

[58] Ketcha J., Dina D.J.D., Ngomo H.M., Ndi N.J. (2012). Preparation and Characterization of Activated Carbons Obtained from Maize Cobs by Zinc Chloride Activation. Am. Chem. Sci. J..

[59] Kumar A., Jena H.M. (2016). Preparation and characterization of high surface area activated carbon from Fox nut (Euryale ferox) shell by chemical activation with H3PO4. Results Phys..

[60] Shi G., Liu C., Wang G., Chen X., Li L., Jiang X., Zhang P., Dong Y., Jia S., Tian H. (2018). Preparation and electrochemical performance of electrospun biomass-based activated carbon nanofibers. Ionics.

[61] Moulder J.F., Stickle W.F., Sobol P.E., Bomben K.D. (1992). Handbook of X-ray Photoelectron Spectroscopy.

[62] Ternero-Hidalgo J.J., Rosas J.M., Palomo J., Valero-Romero M.J., Rodríguez-Mirasol J., Cordero T. (2016). Functionalization of activated carbons by HNO3 treatment: Influence of phosphorus surface groups. Carbon.

[63] Hasegawa G., Deguchi T., Kanamori K., Kobayashi Y., Kageyama H., Abe T., Nakanishi K. (2015). High-Level Doping of Nitrogen, Phosphorus, and Sulfur into Activated Carbon Monoliths and Their Electrochemical Capacitances. Chem. Mater..

[64] Perumbilavil S., Sankar P., Rose T.P., Philip R. (2015). White light Z-scan measurements of ultrafast optical nonlinearity in reduced graphene oxide nanosheets in the 400–700 nm region. Appl. Phys. Lett..

[65] Rajkumar M., Hsu C.-T., Wu T., Chen M.-G., Hu C. (2015). Advanced materials for aquous supercapacitors in the asymmetric design. Prog. Nat. Sci. Mater. Int..

[66] Bello A., Sanni D., Adeniji S., Anye V., Orisekeh K., Kigozi M., Koech R. (2020). Combustion synthesis of battery-type positive electrodes for robust aqueous hybrid supercapacitor. J. Energy Storage.

[67] Kigozi M., Koech R.K., Kingsley O., Ojeaga I., Tebandeke E., Kasozi G.N., Onwualu A.P. (2020). Synthesis and characterization of graphene oxide from locally mined graphite flakes and its supercapacitor applications. Results Mater..

[68] Liang Y., Liang F., Zhong H., Li Z., Fu R., Wu D. (2013). An advanced carbonaceous porous network for high-performance organic electrolyte supercapacitors. J. Mater. Chem. A.

[69] Moyo B., Momodu D., Fasakin O., Bello A., Dangbegnon J., Manyala N. (2017). Electrochemical analysis of nanoporous carbons derived from activation of polypyrrole for stable supercapacitors. J. Mater. Sci..

[70] Mathis T.S., Kurra N., Wang X., Pinto D., Simon P., Gogotsi Y. (2019). Energy Storage Data Reporting in Perspective—Guidelines for Interpreting the Performance of Electrochemical Energy Storage Systems. Adv. Energy Mater..

[71] Zhong C., Deng Y., Hu W., Qiao J., Zhang L., Zhang J. (2015). A review of electrolyte materials and compositions for electrochemical supercapacitors. Chem. Soc. Rev..

[72] Bello A., Barzegar F., Madito M.J., Momodu D., Khaleed A., Masikhwa T.M., Dangbegnon J.K., Manyala N. (2016). Electrochemical performance of polypyrrole derived porous activated carbon-based symmetric supercapacitors in various electrolytes. RSC Adv..

[73] Bello A., Barzegar F., Madito M.J., Momodu D., Khaleed A., Olaniyan O., Masikhwa T.M., Dangbegnon J.K., Manyala N. (2017). Floating of PPY Derived Carbon Based Symmetric Supercapacitor in Alkaline Electrolyte. ECS Trans..

[74] Ruch P., Cericola D., Foelske-Schmitz A., Kötz R., Wokaun A. (2010). Aging of electrochemical double layer capacitors with acetonitrile-based electrolyte at elevated voltages. Electrochim. Acta.

[75] Laheaar A., Przygocki P., Abbas Q., Beguin F. (2015). Appropriate methods for evaluating the efficiency and capacitive behavior of different types of supercapacitors. Electrochem. Commun..

[76] Lehtimäki S., Railanmaa A., Keskinen J., Kujala M., Tuukkanen S., Lupo D. (2017). Performance, stability and operation voltage optimization of screen-printed aqueous supercapacitors. Sci. Rep..

[77] Li H., Kang Z., Liu Y., Lee S.-T. (2012). Carbon nanodots: Synthesis, properties and applications. J. Mater. Chem..

[78] Andreas H.A. (2015). Self-Discharge in Electrochemical Capacitors: A Perspective Article. J. Electrochem. Soc..

[79] Oickle A.M. (2013). A Systematic Study of Self-Discharge Mechanisms in Carbon-Based, Aqueous Electrolyte Electrochemical Capacitors. Ph.D. Thesis.

[80] Momodu D., Okafor C., Manyala N., Bello A., ZebazeKana M.G., Ntsoenzok E. (2019). Transformation of Plant Biomass Waste into Resourceful Activated Carbon Nanostructures for Mixed-Assembly Type Electrochemical Capacitors. Waste Biomass Valoriz..

