# Supplementary material for: Modified Activation Process for Supercapacitor Electrode Materials from African Maize Cob
Source: Materials (Basel). 2020 Nov 27;13(23):5412. doi: 10.3390/ma13235412 (PMC7731031; doi:10.3390/ma13235412)
Supplement: Supplementary file 1 [file materials-13-05412-s001.pdf]

materials

Suplementary Materials: Modified Activation Process for Supercapacitor Electrode Materials from African Maize Cob

Moses Kigozi [1,2,*], Ravi Kali [3], Abdulhakeem Bello [1], Balaji Padya [3], Godwin Mong Kalu-Uka [1,4], John Wasswa [5], Pawan Kumar Jain [3], Peter Azikiwe Onwualu [1] and Nelson Yaw Dzade [1,6,*]

1 Department of Materials Science and Engineering, African University of Science and Technology, Km 10 Airport Road, Galadimawa, P.O BOX 681 Garki Abuja Nigeria; mkigozi@aust.edu.ng (M.K.); abello@aust.edu.ng (A.B.) aonwualu@aust.edu.ng (P.A.O.)
2 Department of Chemistry, Faculty of Science and Education, Busitema University, Tororo P.O BOX 236, Uganda
3 Centre for Carbon Materials, International Advanced Research Centre for Powder Metallurgy & New Materials (ARCI), Balapur P.O Hyderabad 500 005, India; nanoravi09@gmail.com (R.K.); balajipadya@gmail.com (B.P.); pkjain@arci.res.in (P.K.J.)
4 Department of Mechanical Engineering, Alex Ekwueme Federal University, Ndufu-Alike, Ebonyi State, P.O. Box 1010, Abakaliki, Nigeria; gkaluuka@aust.edu.ng (G.M.K.-U.)
5 Department of Chemistry, College of Natural Sciences, Makerere University, P. O. Box 7062 Kampala, Uganda; jnwasswa@chemistry.mak.ac.ug
6 School of Chemistry, Cardiff University, Main Building, Park Place, Cardiff CF10 3AT, UK
* Correspondence: mkigozi@aust.edu.ng (M.K.); dzadeny@cardiff.ac.uk (N.Y.D.)

List of Tables

Table S1. The residual mass of raw material powder of maize corn cobs, AC-S-600, AC-S-700, and AC-S-800 activated carbon materials at the different temperature range.

Temperature Range (°C)	Residual Mass (%)			
	Before activation	After Activation		
	Raw material	AC-S-600	AC-S-700	AC-S-800
28–120	5.0	5.0	10.5	8.0
120–200	2.6	2.5	2.4	2.0
200–350	52.4	0.5	1.8	1.2
350–600	14.5	6.0	5.3	4.4
600–1000	10.5	26.2	12.9	11.9
Total loss	85	59.8	67.1	72.5

Table S2. FTIR peaks assignment for functionality/groups on AC surfaces for AC-S-600, AC-S-700, and AC-S-800 materials.

Wavenumber (cm^{-1})	Functionality/Groups	Transmittance Strength
1000–1500	C–OH stretching<br>C–O stretching (ethers)<br>C–N<br>Nitro groups<br>Aromatic compound	Strong/Medium
1540	Quinones (Carboxylic acid)	Weak
1743	C=O stretching (Carboxylic acid, Lactones, carbonyl groups)	Strong
2369	Methyl and methylene groups	Weak
2932–3000	C–H stretching (CH_3–,–CH_2–, carboxylic acid)	Weak
3459–4000	O–H overtones (carboxylic acid/phenolic group)	Weak

Table S3. Boehm Acidic and Basic surface characterization of the activated carbon materials.

AC Sample	Acidic Functional Groups			Total Acidity (mmol/g)	Total Basicity (mmol/g)
	Carboxylic (mmol/g)	Lactonic (mmol/g)	Phenolic (mmol/g)		
AC-S-600	0.250	0.168	0.022	0.440	0.090
AC-S-700	0.244	0.02	0.20	0.464	0.118
AC-S-800	0.112	0.100	0.230	0.442	0.420

Table S4. XPS mass surface concentration of activated carbon material's composition (%), binding energy position and their Full Width at Half Maximum intensity (FWHM).

AC Samples	Elementals Peaks	Composition (%)	Position (eV)	FWHM (eV)
AC-S-600	C_{1s}	67.01	283.7	1.903
–	O_{1s}	22.59	531.7	2.636
–	S_{2p}	2.60	168.7	2.618
–	B_{1s}	5.38	191.7	2.272
–	Na_{1s}	2.42	1071.7	2.148
–	–	–	–	–
AC-S-700	C_{1s}	56.97	283.7	2.515
–	O_{1s}	29.90	532.7	2.746
–	S_{2p}	2.12	169.7	2.413
–	B_{1s}	5.97	192.7	1.968
–	Na_{1s}	5.04	1071.7	2.485
–	–	–	–	–
AC-S-800	C_{1s}	56.45	283.7	2.294
–	O_{1s}	31.70	531.7	2.700
–	S_{2p}	2.71	168.7	2.759
–	B_{1s}	4.09	192.7	2.469
–	Na_{1s}	5.05	1071.7	2.360

Table S5. XPS spectra of AC-S-600, AC-S-700, and AC-S-800 activated materials, Binding Energy, Functional group assignment and their relative chemical bonding contents.

Peaks	Binding Energy (eV)	Functional Group Assignments	Samples % Content		
			AC-S-600	AC-S-700	AC-S-800
O_{1s}	530.8–531.1	C=O, O_2,–O–, O–CH_2C–*	–	–	8.55
–	531.6–531.8	C=O, O_2, S=O, O–C_6H_5NH*,	91.61	–	74.82
–	532.2–533.0	C–O, –O– (C–O–S)	8.39	100	11.43
–	535.8–535.9	C–O, O_2	–	–	5.20
–	–	–	–	–	–
C_{1s}	283.7–283.9	–C–C–, C=O,	73.22	–	69.74
–	284.1–284.6	–C–C–, C=O, C–O–S	–	39.44	-
–	284.7–285.4	C–C, C–H, C_g C=C–C*	18.06	28.39	20.57
–	285.5–288.6	C–O, C=O, O–C=O, C–O–C, S=C=S*	5.17	23.48	–
–	288.7–289.7	O=C=S (OC*S)*	3.55	8.69	9.69
–	–	–	–	–	–
S_{2p}	168.4–168.9	C–O–S, O=S=O*	62.82	59.07	86.01
–	169.6–170.1	S=O, O=S=O*	37.18	40.93	13.99

*https://srdata.nist.gov/xps/ElmComposition.aspx was also used for assigning groups.

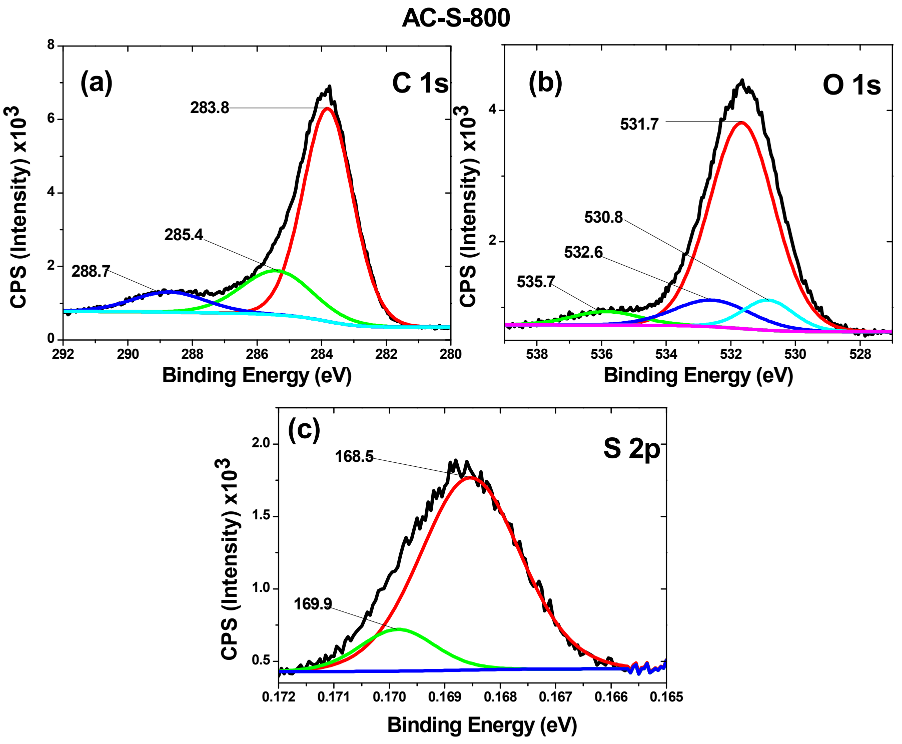

(i)

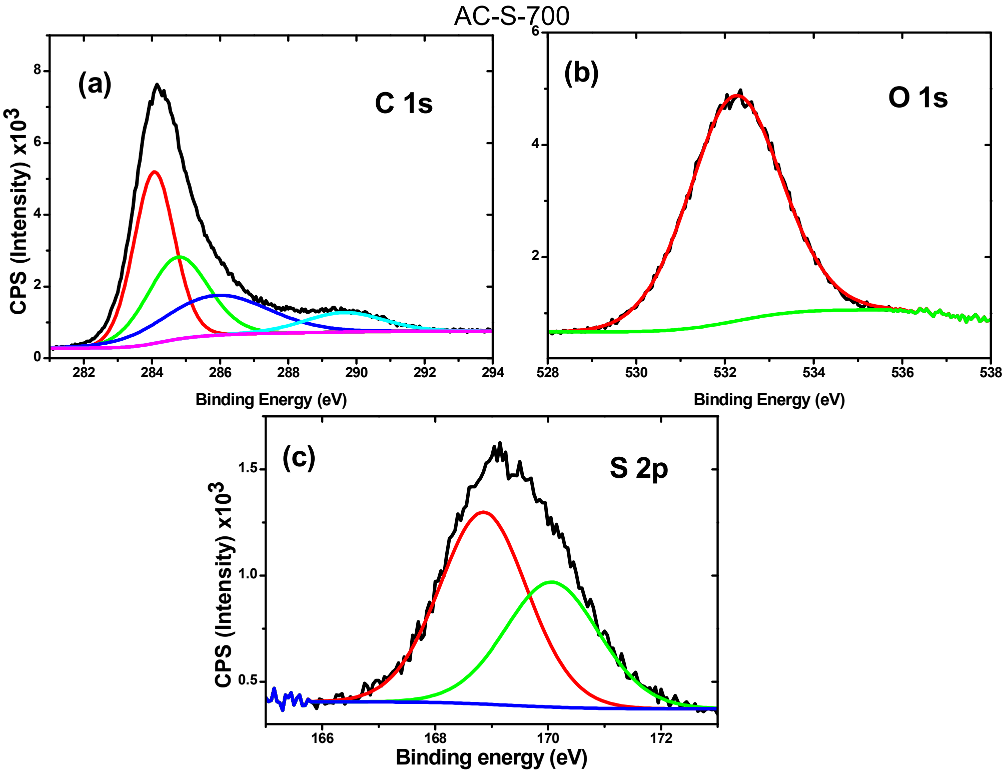

(ii)

Figure S1. (**i**) XPS deconvoluted spectra (**a**) carbon, (**b**) oxygen and (**c**) Sulphur for AC-S-800 activated carbon material. (**ii**). XPS deconvoluted spectra (**a**) carbon, (**b**) oxygen and (**c**) Sulphur for AC-S-700 activated carbon material.

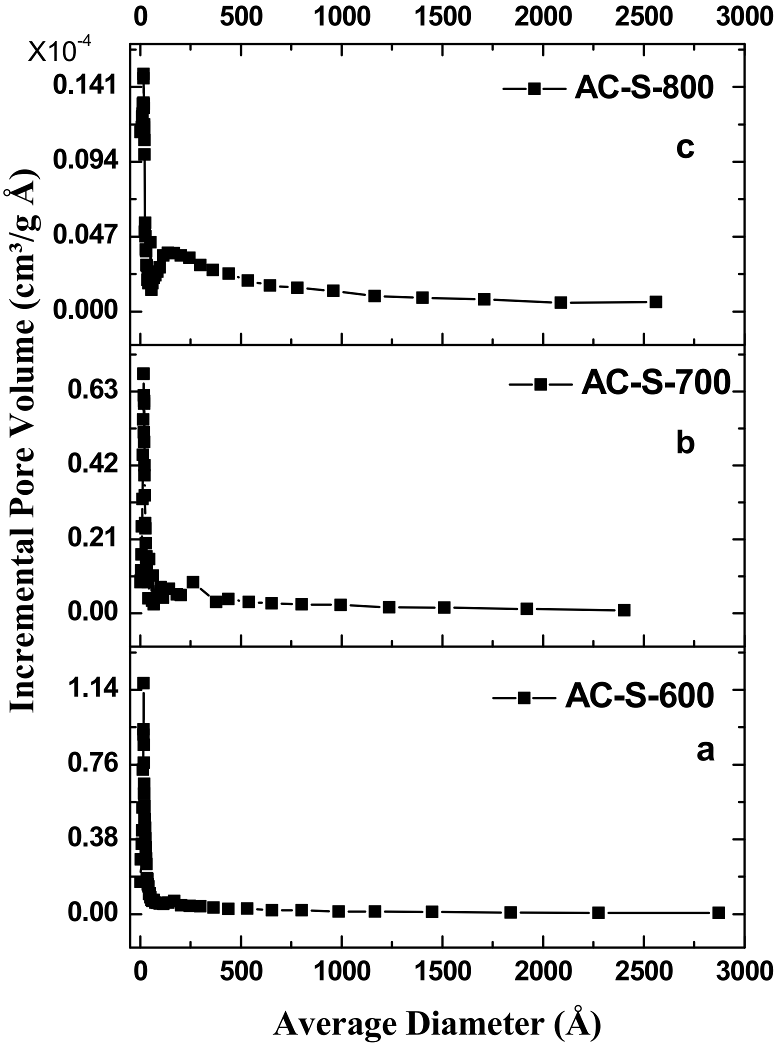

Figure S2. Pole size distribution for (**a**) AC-S-600, (**b**) AC-S-700, and (**c**) AC-S-800 activated carbon material samples.

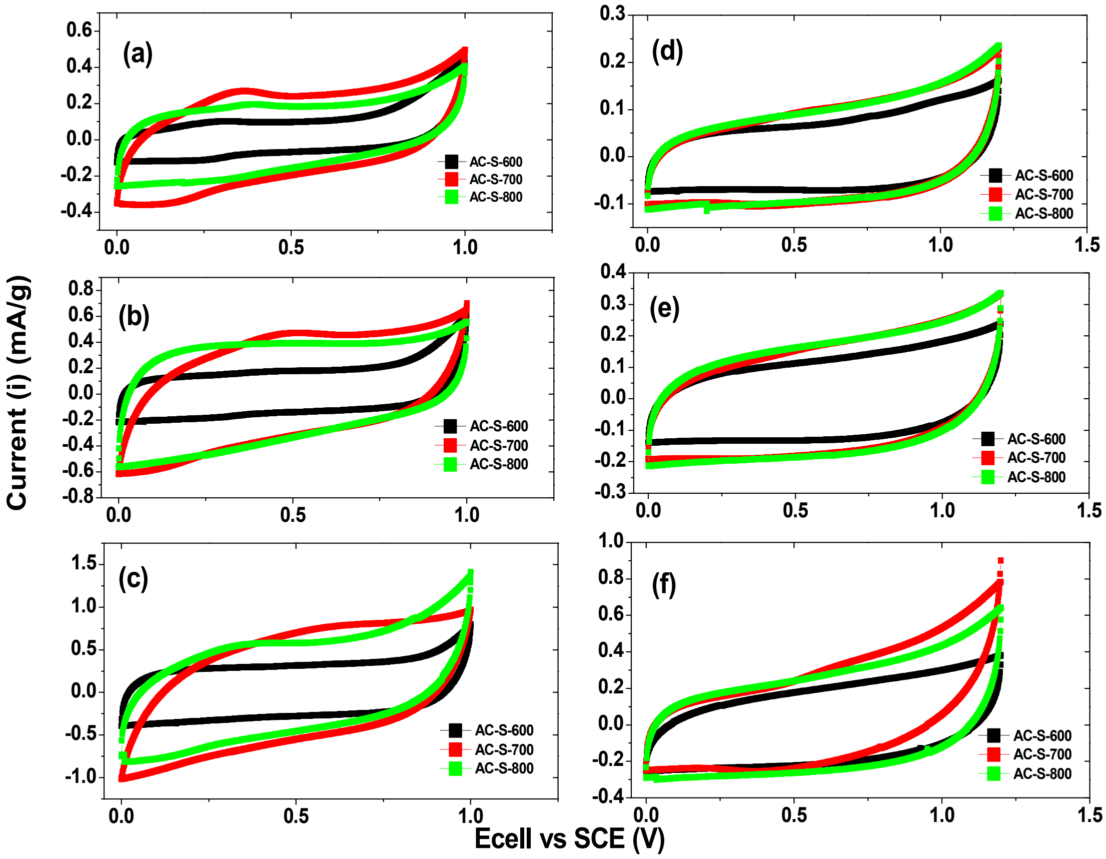

Figure S3. Combined CV curves of AC-S-Activated carbon samples at scan rates of; 5 mV/s (**a** & **d**), 10 mV/s (**b** & **e**) and 20 mV/s (**c** & **f**) with 6M KOH (**a**–**c**) and 1M Na_2SO_4 (**d**–**f**) electrolyte.

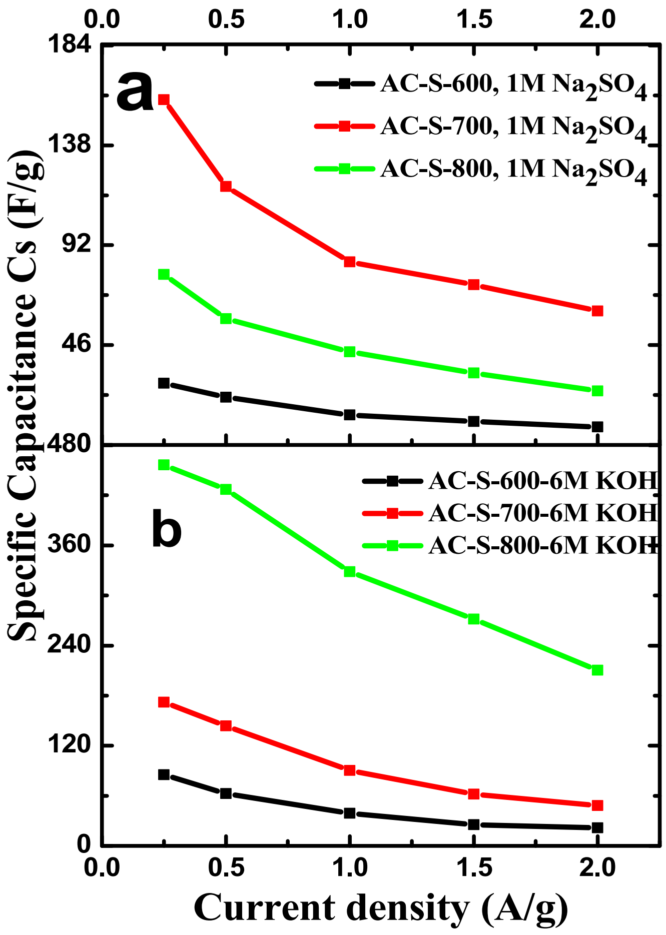

Figure S4. Specific Capacitance (F/g) with current density (A/g) curves plot for AC-S-600, AC-S-700, and AC-S-800 for two different electrolytes (6M KOH and 1M Na2SO4).

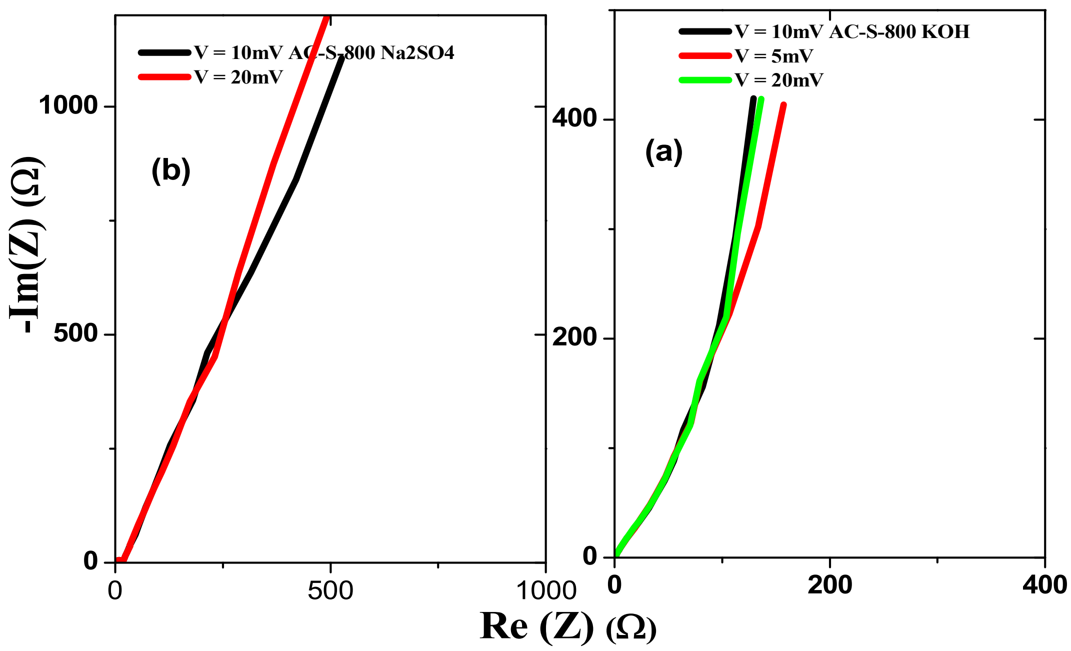

Figure S5. EIS Nyquist plots for AC-S-800 electrode material at different potentials using different electrolytes (**a**) 6M KOH at 5mV, 10mV and 20mV, (**b**)1M Na2SO4 at 10mV and 20mV.

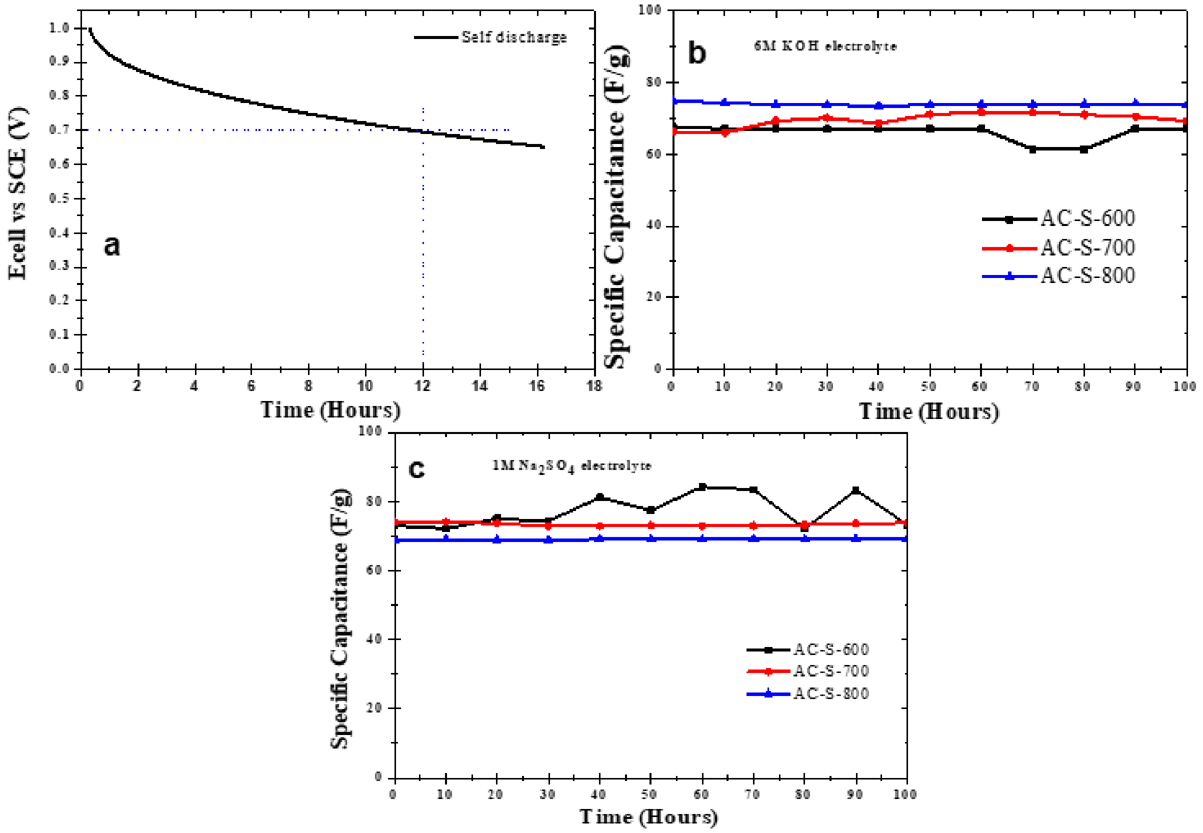

Figure S6. (**a**) The self-discharge of the device with the highest capacitance assembled with AC-S-800 with 6M KOH after charging to 1.0V, (**b**) Voltage holding stability capacitances of AC-S-600, AC-S-700, and AC-S-800 with 6M KOH electrolyte at the current density of 1.0.A/g for 100 h, (**c**) stability of AC-S-600, AC-S-700, and AC-S-800 with 1M Na_2SO_4 electrolyte at the current density of 1.0 A/g with 3 charges/discharge cycles and charge hold for 10 h then repeated for 100 hours.

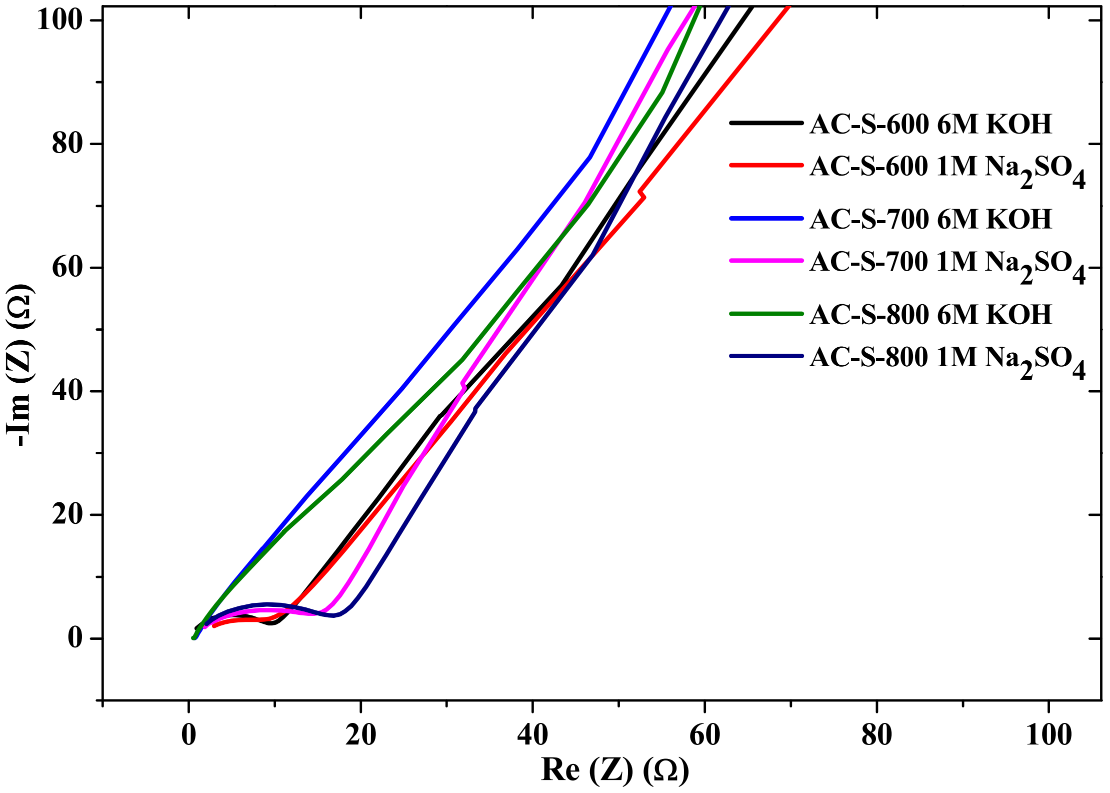

Figure S7. Electrochemical Impedance Spectroscopy (EIS) Nyquist plot for AC-S-600, AC-S-700, and AC-S-800 for two different electrolytes (6M KOH and 1M Na_2SO_4) at the potential of 10mV at low frequency.

© 2020 by the authors. Licensee MDPI, Basel, Switzerland. This article is an open access article distributed under the terms and conditions of the Creative Commons Attribution (CC BY) license (http://creativecommons.org/licenses/by/4.0/).
